# Aldehyde metabolism governs resilience of mucociliary clearance to air pollution exposure

**DOI:** 10.1172/JCI191276

**Published:** 2025-05-15

**Authors:** Noriko Shinjyo, Haruna Kimura, Tomomi Yoshihara, Jun Suzuki, Masaya Yamaguchi, Shigetada Kawabata, Yasutaka Okabe

**Affiliations:** 1 Laboratory of Immune Homeostasis, World Premier International Research Center Initiative, Immunology Frontier Research Center (IFReC), The University of Osaka, Osaka, Japan.; 2 School of Tropical Medicine and Global Health, Nagasaki University, Nagasaki, Japan.; 3 Graduate School of Biostudies and; 4 Institute for Integrated Cell-Material Sciences, Kyoto University, Kyoto, Japan.; 5 Center for Integrated Biosystems, Institute of Biomedical Sciences, Academia Sinica, Taipei, Taiwan.; 6 Core Research for Evolutional Science and Technology (CREST), Japan Science and Technology Agency, Kawaguchi, Japan.; 7 Microbial Research Center for Health and Medicine, National Institutes of Biomedical Innovation, Health and Nutrition, Osaka, Japan.; 8 Department of Microbiology, Graduate School of Dentistry, and; 9 Center for Infectious Disease Education and Research (CiDER), The University of Osaka, Osaka, Japan.

**Keywords:** Cell biology, Infectious disease, Public Health, Bacterial infections, Homeostasis, Toxicology

## Abstract

Air pollution is a serious environmental threat to public health; however, the molecular basis underlying its detrimental effects on respiratory fitness remains poorly understood. Here, we showed that exposure to particulate matter ≤ 2.5 μm (PM_2.5_), a substantial fraction of air pollutants, induced the generation of reactive aldehyde species in the airway. We identified aldehyde dehydrogenase 1A1 (ALDH1A1), which was selectively expressed in airway epithelium, as an enzyme responsible for detoxifying these reactive aldehyde species. Loss of ALDH1A1 function resulted in the accumulation of aldehyde adducts in the airway, which selectively impaired mucociliary clearance (MCC), a critical defense mechanism against respiratory pathogens. Thus, ALDH1A1-deficient mice pre-exposed to PM_2.5_ exhibited increased susceptibility to pneumonia. Conversely, pharmacological enhancement of ALDH1A1 activity promoted the restoration of MCC function. These findings elucidate the critical role of aldehyde metabolism in protecting against PM_2.5_ exposure, offering a potential target to mitigate the negative health consequences of air pollution.

## Abstract 2

Aldehyde metabolism plays a crucial role in protecting cilia during exposure to air pollution.

## Introduction

Most urban residents around the world breathe unhealthy levels of air pollution (1). Epidemiological studies demonstrated an association between ambient air pollution and increasing mortality and morbidity (2, 3). Particulate matter ≤ 2.5 μm (PM_2.5_) is a major hazardous component of air pollutants, primarily generated from the combustion of gasoline, oil, diesel fuel, or wood products. It is a leading driver of multiple adverse health effects, including respiratory diseases, lung cancer, and heart diseases, with an estimated annual global mortality of up to 8.9 million deaths (4–6). Consequently, PM_2.5_ is considered one of the largest environmental threats to public health (7, 8).

The airway serves as the first line of defense in the respiratory system, functioning not only as a physical barrier but also actively eliminating inhaled pathogens and particles through mucociliary clearance (MCC) (9). MCC consists of 2 essential components: mucus secreted by goblet cells and submucosal glands, which entraps pathogens and particles, and ciliated cells, which provide the force necessary for mucus movement by coordinated and rhythmic beating of their cilia (10–12). This collaborative function of mucus and cilia facilitates the transport of pathogens and particles toward the pharynx, where they are eliminated by swallowing. Although MCC is crucial for maintaining the sterility of the respiratory tract, environmental pollutants such as ultrafine particles, fine particles, ozone, nitrogen oxides, and transition metals can reach deep into the respiratory tract before being captured by mucus (13, 14). Many of these pollutants are potent oxidants or can generate ROS, which trigger oxidative stress and contribute to the initial pathophysiology (15, 16). For instance, exposure to PM_2.5_ triggers oxidative airway injury, leading to ciliary damage and impaired MCC, which increases susceptibility to respiratory infections (17). Indeed, both long- and short-term exposure to PM_2.5_ have been linked to an increased incidence of pneumonia, contributing to approximately 7% of deaths associated with PM_2.5_ exposure (18, 19). The ability to restore MCC function is crucial for mitigating the negative consequences of PM_2.5_ exposure (20); however, the mechanisms underlying cilia regeneration following oxidative airway injury remain largely unknown.

Each airway ciliated cell is equipped with 200–300 cilia with diameters ranging from 0.2 to 0.3 μm and lengths of 4–7 μm, which are contiguous with the plasma membrane (11). These cilia are uniformly oriented toward the luminal surface of the respiratory tract, leading to a remarkable expansion of the plasma membrane, which is estimated to be hundreds of times larger than that of typical cell types. This extensive surface area makes ciliated cells a major point of contact with environmental pollutants. Membrane phospholipids and triglycerides are susceptible to oxidative stress, and oxidation of polyunsaturated fatty acids (PUFAs) in membrane lipid bilayers generates α,β-unsaturated aldehyde species, including acrolein and 4-hydroxy-2-nonenal (4-HNE), as by-products (21–23). These unsaturated aldehydes are highly reactive and target diverse nucleophiles, including proteins and DNA, forming aldehyde adducts that impair cellular function. Given their structural features, ciliated cells may be especially vulnerable to reactive aldehydes generated by air pollutants.

Here, we showed that PM_2.5_ induced the formation of reactive aldehyde species in the airway. We found that aldehyde dehydrogenase 1A1 (ALDH1A1), which was selectively expressed in airway epithelium, was essential for degrading these aldehydes. We also observed that absence of ALDH1A1 led to aberrant cilia regeneration upon PM_2.5_ exposure, resulting in impaired MCC and increased susceptibility to *Streptococcus pneumoniae* infection. Lastly, we revealed that enhancement of endogenous ALDH activity by the administration of a small-molecule ALDH1A1 activator facilitated MCC restoration. Altogether, these results demonstrate that aldehyde metabolism ensures ciliary resilience, illuminating its therapeutic potential in mitigating respiratory disorders associated with air pollution.

## Results

### PM_2.5_ induces the formation of reactive aldehyde species in the airway.

**Figure 1:**
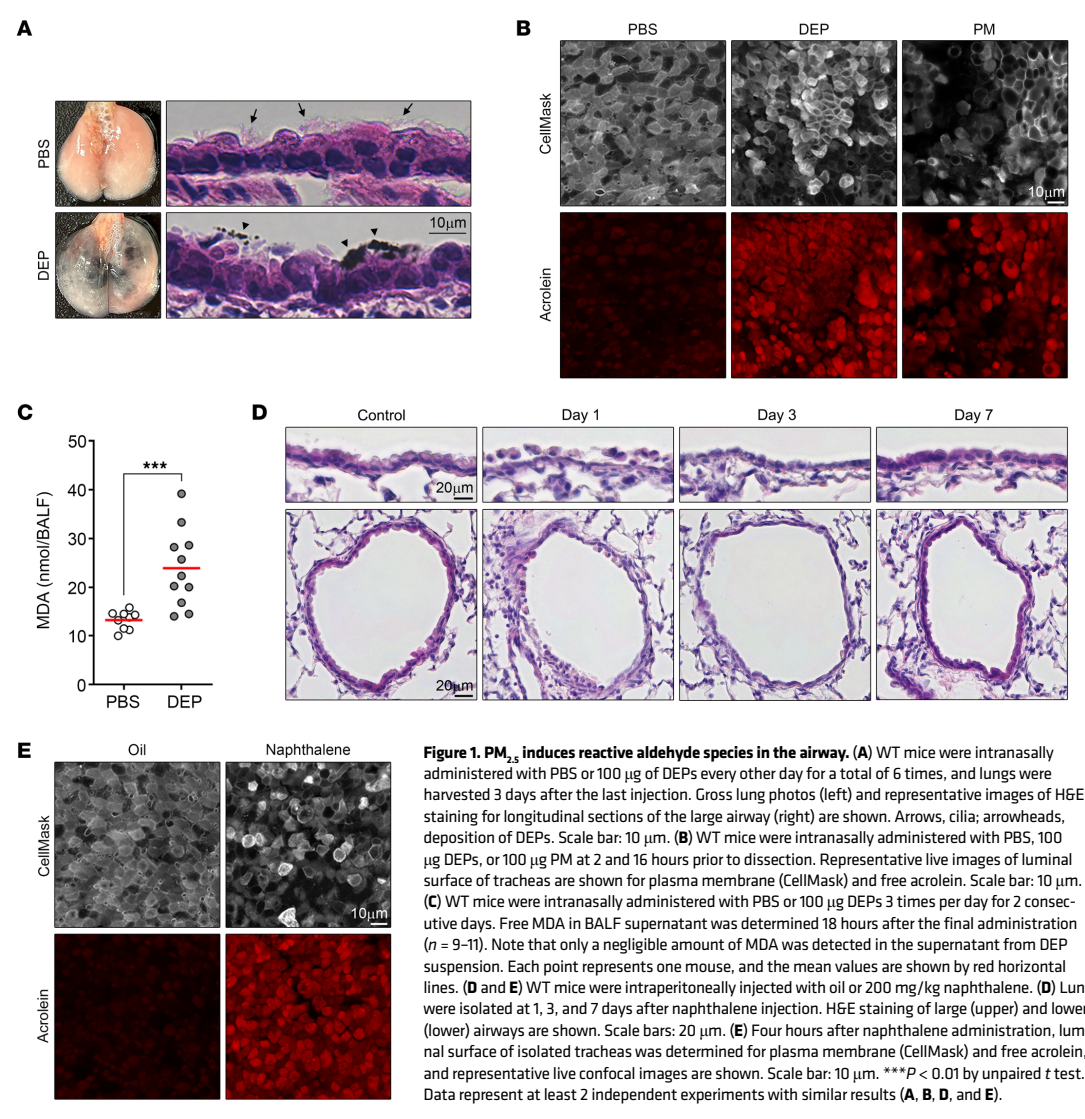
PM_2.5_ induces reactive aldehyde species in the airway. (**A**) WT mice were intranasally administered with PBS or 100 μg of DEPs every other day for a total of 6 times, and lungs were harvested 3 days after the last injection. Gross lung photos (left) and representative images of H&E staining for longitudinal sections of the large airway (right) are shown. Arrows, cilia; arrowheads, deposition of DEPs. Scale bar: 10 μm. (**B**) WT mice were intranasally administered with PBS, 100 μg DEPs, or 100 μg PM at 2 and 16 hours prior to dissection. Representative live images of luminal surface of tracheas are shown for plasma membrane (CellMask) and free acrolein. Scale bar: 10 μm. (**C**) WT mice were intranasally administered with PBS or 100 μg DEPs 3 times per day for 2 consecutive days. Free MDA in BALF supernatant was determined 18 hours after the final administration (*n* = 9–11). Note that only a negligible amount of MDA was detected in the supernatant from DEP suspension. Each point represents one mouse, and the mean values are shown by red horizontal lines. (**D** and **E**) WT mice were intraperitoneally injected with oil or 200 mg/kg naphthalene. (**D**) Lungs were isolated at 1, 3, and 7 days after naphthalene injection. H&E staining of large (upper) and lower (lower) airways are shown. Scale bars: 20 μm. (**E**) Four hours after naphthalene administration, luminal surface of isolated tracheas was determined for plasma membrane (CellMask) and free acrolein, and representative live confocal images are shown. Scale bar: 10 μm. ***P < 0.01 by unpaired *t* test. Data represent at least 2 independent experiments with similar results (**A**, **B**, **D**, and **E**).

To investigate the mechanisms by which PM_2.5_ induces airway damage and disrupts respiratory homeostasis, we employed intranasal delivery of diesel exhaust particles (DEPs), which constitute approximately 20% of PM_2.5_ in ambient air (24). In accordance with previous studies (25–30), intranasal administration of 100 μg DEPs was performed every other day for a total of 6 times. This resulted in the accumulation of DEPs in the respiratory tract, leading to airway epithelial damage, including ciliary loss (Figure 1A). In our investigation of tissue damage biomarkers, we found that DEPs led to de novo generation (free form) of acrolein, a lipid peroxide–derived aldehyde, in the airway epithelium (Figure 1B). An elevated acrolein level was also observed following intranasal delivery of PM (<4.0 μm) (Figure 1B). Furthermore, DEP exposure increased another lipid peroxide–derived aldehyde, malondialdehyde (MDA), in bronchoalveolar lavage fluid (BALF) (Figure 1C). The increase in these lipid peroxide–derived aldehydes suggests that PM_2.5_ exposure induces oxidation of PUFAs in membrane lipid bilayers. Indeed, we observed that DEPs elevated the level of ROS and lipid peroxidation in the airway epithelium, with variability observed across areas (Supplemental Figure 1, A and B; supplemental material available online with this article; https://doi.org/10.1172/JCI191276DS1). Furthermore, DEPs induced the expression of oxidative stress–responsive genes (*Hmox1* and *Txnrd1*) in the lung (Supplemental Figure 1C), in line with previous observations (31).

Accumulating evidence indicates oxidative stress markedly contributes to the adverse consequence of PM_2.5_ exposure (15, 16), and our results suggest that lipid peroxide–derived aldehydes are involved in airway damage. PM_2.5_ consists of a wide range of chemical components, depending on their sources, with polycyclic aromatic hydrocarbons (PAHs) being major contributors to oxidative stress (32). To specifically interrogate oxidative stress in generating reactive aldehyde species in the airways, we employed naphthalene, the most abundant PAHs found in ambient urban air (33). Naphthalene induces oxidative damage through the formation of naphthalene oxide, mediated by the cytochrome P450 enzyme CYP2F2 (34, 35). Although naphthalene primarily targets club cells (secretory cells) in the airway epithelium, where CYP2F2 is predominantly expressed, previous studies have demonstrated dose-dependent toxicity of naphthalene to other airway epithelial cell types, including ciliated cells (36). Immunofluorescence staining and publicly available mouse single-cell RNA-sequence (scRNA-seq) data further confirmed substantial CYP2F2 expression in ciliated cells (Supplemental Figure 1, D and E). Therefore, we utilized naphthalene exposure as a proxy for oxidative airway injury induced by PM_2.5_ exposure. Intraperitoneal administration of naphthalene caused most of the airway epithelium slough off within 1 d, with substantial regeneration occurring 1 week after administration (Figure 1D). It rapidly increased ROS levels and lipid peroxidation and activated oxidative stress–responsive genes (Supplemental Figure 1, F–H). Furthermore, naphthalene markedly raised acrolein levels in the trachea (Figure 1E). These findings indicate that the generation of reactive aldehyde species in the airways is, at least in part, driven by oxidative stress induced by PM_2.5_ exposure.

### ALDH1A1 degrades reactive aldehyde species in airway.

**Figure 2:**
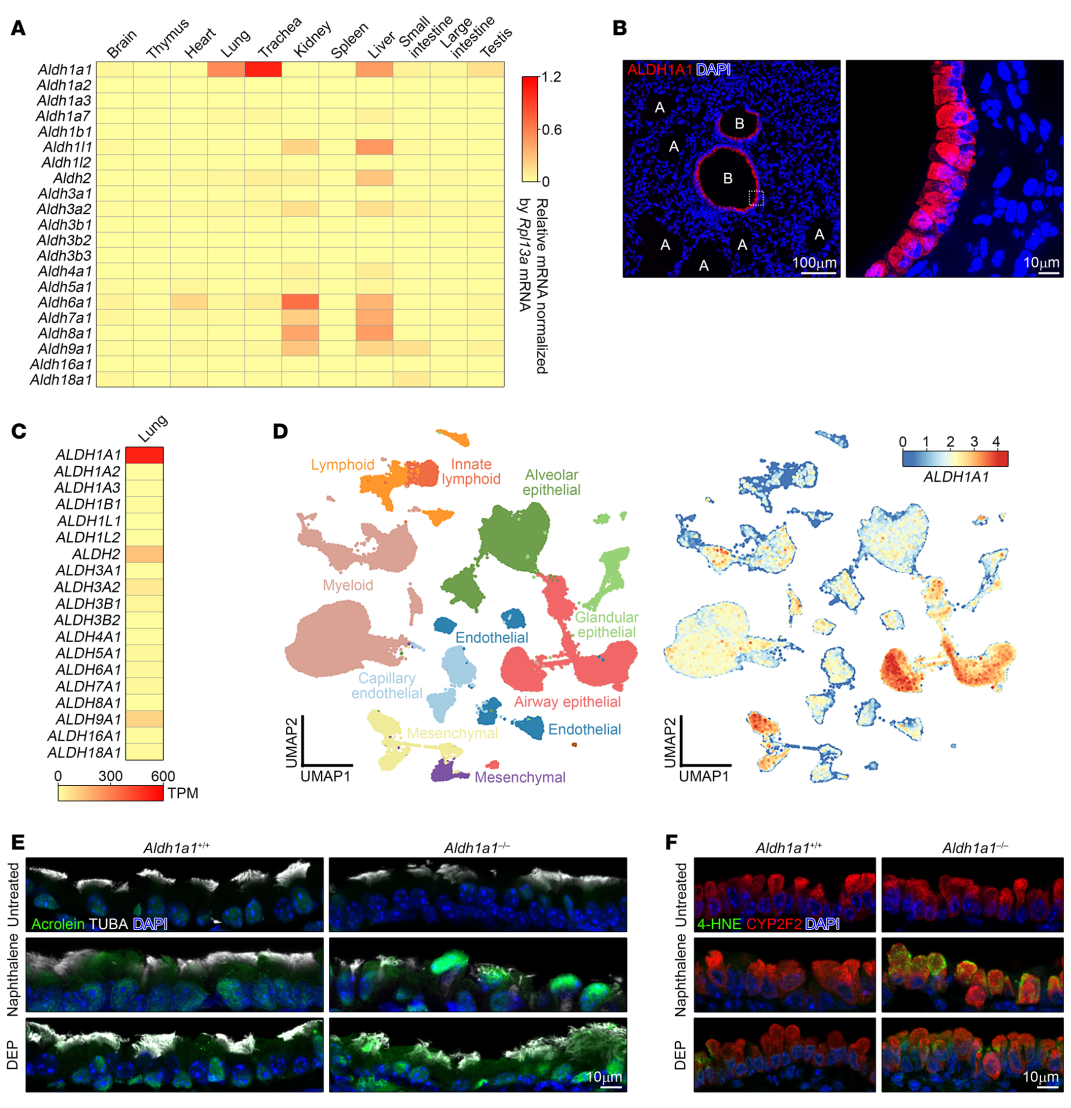
Essential role of ALDH1A1 in detoxifying reactive aldehyde species in the airway. (**A**) Expression of mRNA of mouse ALDH family members in indicated tissues (*n* = 3) was determined by quantitative PCR, and copy numbers per *Rpl13a* mRNA are visualized using a heat map. (**B**) Lung of WT mice was analyzed by immunofluorescence staining for ALDH1A1 and nuclei (DAPI). Higher magnification of bronchiolar epithelium (gated in left panel) is shown in right panel. B, bronchus; A, alveolar duct. Scale bars: 100 μm (left), 10 μm (right). (**C** and **D**) Bulk RNA-seq and scRNA-seq data of human lungs obtained from ENCODE and LungMAP Consortium were analyzed. (**C**) Transcripts per kilobase million (TPM) of ALDH family members are visualized using a heat map. (**D**) Uniform manifold approximation and projection (UMAP) visualization of color-coded human lung cell populations (left) and *ALDH1A1* mRNA projection with highest normalized expression level (right) are shown. (**E** and **F**) *Aldh1a1*^+/+^ and *Aldh1a1*^–/–^ mice were intraperitoneally injected with 200 mg/kg naphthalene at 2-week intervals for a total of 2 times, and lungs were harvested 2 weeks after the second administration. Alternatively, mice were intranasally injected with 100 μg DEPs every other day for a total of 6 times, and lungs were harvested 3 days after the last administration. Immunofluorescence staining of longitudinal sections of the large airway was performed for acrolein adduct, cilia (TUBA), and nuclei (DAPI) (**E**) or 4-HNE adduct, airway epithelial cells (CYP2F2), and nuclei (DAPI) (**F**), and representative images are shown. Scale bars: 10 μm (**E** and **F**). Data represent at least 2 independent experiments with similar results (**B**, **E**, and **F**).

The ALDH superfamily, which comprises 21 functional genes in mice and 19 in human genomes, plays a vital role in protecting against reactive aldehyde species through enzyme catalysis (37). Among ALDH family members, *Aldh1a1* shows the highest mRNA expression in mouse lung and trachea (Figure 2A). ALDH1A1 was selectively expressed in the airway epithelium, particularly in ciliated cells and club cells, but was absent or expressed at low levels in alveolar epithelium and interstitial regions (Figure 2B and Supplemental Figure 1E). Furthermore, publicly available human RNA-seq and scRNA-seq data similarly showed that *ALDH1A1* mRNA is most highly expressed in the lung, where it is predominantly found in airway epithelial cells (38) (Figure 2, C and D). These results indicate that ALDH1A1 is selectively expressed in airway epithelium in both mice and humans.

To examine the role of ALDH1A1 in metabolizing reactive aldehyde species, we generated ALDH1A1-deficient mice by introducing a STOP cassette into the second exon of the *Aldh1a1* gene locus (Supplemental Figure 2A). ALDH1A1-deficient mice showed apparently normal development, including gross histology of the lung and airway (Supplemental Figure 2, B and C), consistent with previous report (39). We conducted immunostaining analysis to assess aldehyde adduct formation at a time point when the initial redox stress induced by DEPs or naphthalene exposure was likely to have subsided (Figure 2, E and F). *Aldh1a1*^+/+^ mice showed minimal deposition of acrolein adducts and 4-HNE adducts in the airways, suggesting efficient metabolizing of reactive aldehyde species in these mice (Figure 2, E and F). In contrast, *Aldh1a1*^–/–^ mice exhibited increased accumulation of aldehyde adducts in the airways. These results indicate that ALDH1A1 plays a crucial role in metabolizing reactive aldehyde species in the airway epithelium.

In addition to *Aldh1a1*, airway epithelial cells are found to express antioxidant genes, including *Glutathione peroxidase 2* (*Gpx2*), *Peroxiredoxin 1* (*Prdx1*), *Prdx6*, *Superoxide dismutase 1* (*Sod1*), and *Sod2* (Supplemental Figure 2D). This suggests that, alongside ALDH1A1, these genes may contribute to mitigating oxidative tissue damage induced by PM_2.5_ exposure.

### ALDH1A1 deficiency causes aberrant cilia formation in response to oxidative injury.

**Figure 3:**
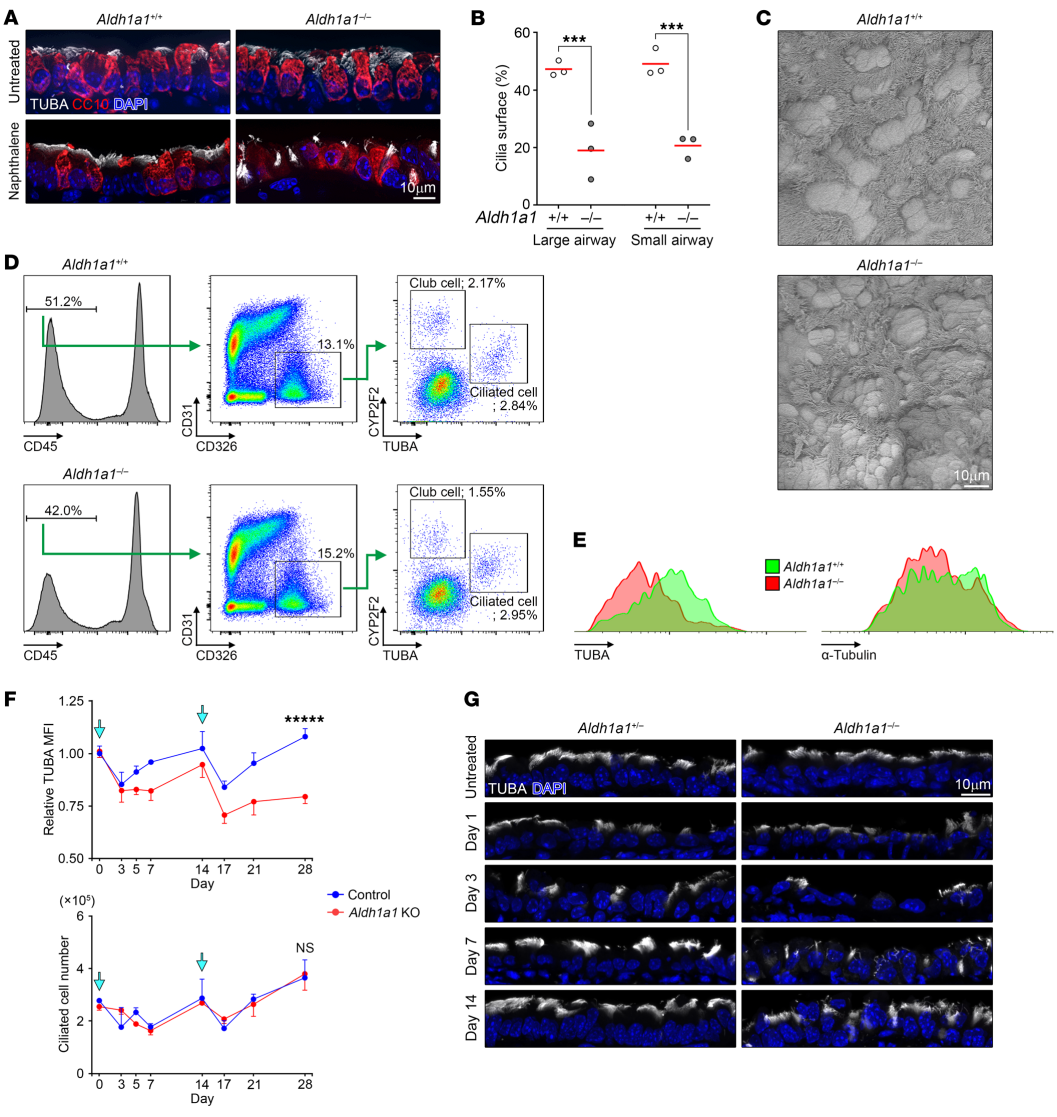
ALDH1A1 deficiency leads to impaired cilia regeneration. (**A**–**E**) Mice with indicated genotypes (control, *Aldh1a1*^+/+^ or *Aldh1a1*^+/–^; KO, *Aldh1a1*^–/–^) were intraperitoneally injected with 200 mg/kg naphthalene at 2-week intervals for a total of 2 times. Lungs were harvested 2 weeks after the second injection. Scale bars: 10 μm (**A**, **C**, and **G**). (**A**) immunofluorescence staining of longitudinal sections of the large airway was performed for cilia (TUBA), airway epithelial cells (CC10), and nuclei (DAPI), and representative images are shown. (**B**) The percentage of ciliated surface of large and small airway epithelium in (**A**) and Supplemental Figure 3A are shown (*n* = 3). (**C**) Representative scanning electron microscopy images of the naphthalene-exposed large airway epithelium are shown. (**D**) Flow cytometry analysis of lung cells in naphthalene-exposed mice was conducted. (**E**) TUBA and total α-tubulin levels of ciliated cells in (**D**) is shown. (**F** and **G**) Mice with indicated genotypes were intraperitoneally injected with 200 mg/kg naphthalene. (**F**) Mean fluorescence intensity (MFI) of TUBA levels (left) and number of lung ciliated cells (right) were determined at specified days after administration, and the mean values with SEM are shown (*n* = 3–9). The days of naphthalene injection are indicated by light blue arrows. (**G**) At specified days after administration, immunofluorescence staining of longitudinal sections of the large airway was performed for cilia (TUBA) and nuclei (DAPI). Each point represents one mouse, and the mean values are shown by red horizontal lines (**B**). *****P < 0.0001, ***P < 0.01, and NS, not significant by unpaired *t* test. Data represent at least 3 independent experiments with similar results (**A**, **C**–**E**, and **G**).

Given the harmful effects of reactive aldehyde species, we next assessed the impact of ALDH1A1 deficiency on respiratory function. Since DEP exposure causes variable damage across different regions of the airways (Figure 1A and Supplemental Figure 1B), making it challenging to accurately assess overall airway obstruction, we focused on naphthalene exposure, which induced more uniform airway injury. In the evaluation of airway epithelial cell markers, we found that naphthalene-exposed ALDH1A1-deficient mice exhibited disorganized arrangement and orientation of cilia, which was demonstrated by staining for a structural component of the cilium, acetylation of α-tubulin at lysine-40 (TUBA) (Figure 3A). Similar disorganization of cilia was also found in ALDH1A1-deficient mice exposed to DEPs (Figure 2E). In naphthalene-exposed *Aldh1a1*^–/–^ mice, we observed a reduction in the ciliary area on the luminal surface of both large and small airways (Figure 3B and Supplemental Figure 3A). The impaired cilia formation was further confirmed by vertical imaging of scanning electron microscopy (Figure 3C).

We next assessed the quantification of TUBA levels in ciliated cells using flow cytometry. We found naphthalene exposure resulted in a substantial reduction in TUBA levels in *Aldh1a1*^–/–^ ciliated cells, while total α-tubulin remained unaffected (Figure 3, D and E, and Supplemental Figure 3B). To examine whether ALDH1A1 deficiency exacerbates initial airway damage or impairs ciliary regeneration, we analyzed the kinetics of the reduction in TUBA levels. At the initial phase of airway injury, both control and *Aldh1a1*^–/–^ mice showed a similar reduction in TUBA levels, which peaked 3 days after naphthalene exposure (Figure 3, F and G). In contrast, *Aldh1a1*^–/–^ mice showed a delayed recovery in TUBA levels, which was further exacerbated by a second naphthalene administration (Figure 3, F and G). This suggests that repetitive oxidative airway injury impairs ciliary regeneration more severely in the absence of ALDH1A1. Notably, there was no significant difference in the number of ciliated cells and club cells between control and *Aldh1a1*^–/–^ mice during naphthalene exposure (Figure 3F and Supplemental Figure 3C). Additionally, no significant differences were observed in the expression of key airway epithelial cell markers, including *Foxj1* (ciliated cells), *Scgb1a1* (club cells), *Muc5ac* (goblet cells), *Trp63* (basal cells), and *Krt13* (squamous cells), in the trachea of control and *Aldh1a1*^–/–^ mice (Supplemental Figure 3D). These findings suggest that ALDH1A1 deficiency selectively impairs ciliary regeneration following oxidative airway injury, without affecting the overall population of airway epithelial cells.

Apart from its role in detoxifying harmful aldehydes, ALDH1A1 also facilitates the conversion of retinaldehyde into retinoic acid (RA), which functions as a ligand for retinoic acid nuclear receptors (RARs) to induce the gene expression program and regulate tissue repair (40–42). Nonetheless, lung RNA-seq analysis of *Aldh1a1*^+/+^ and *Aldh1a1*^–/–^ mice showed that ALDH1A1 deficiency did not lead to a notable alteration in the overall pattern of gene expression (Supplemental Figure 3E). Pathway analysis identified significant enrichment for the terms “cilium movement” and “axoneme assembly” (*P* < 0.05), while “RAR target genes” did not exhibit significant enrichment (Supplemental Figure 3F). Furthermore, naphthalene exposure did not result in a notable change in the expression of putative RA-responsive genes, as well as members of ALDH family and antioxidant genes (Supplemental Figure 3G). Collectively, these results suggest that ALDH1A1 deficiency primarily leads to cilia impairment, while exerting minimal influence on global gene expression, including RA-responsive genes.

### Aberrant cilia regeneration in ciliated cell culture.

**Figure 4:**
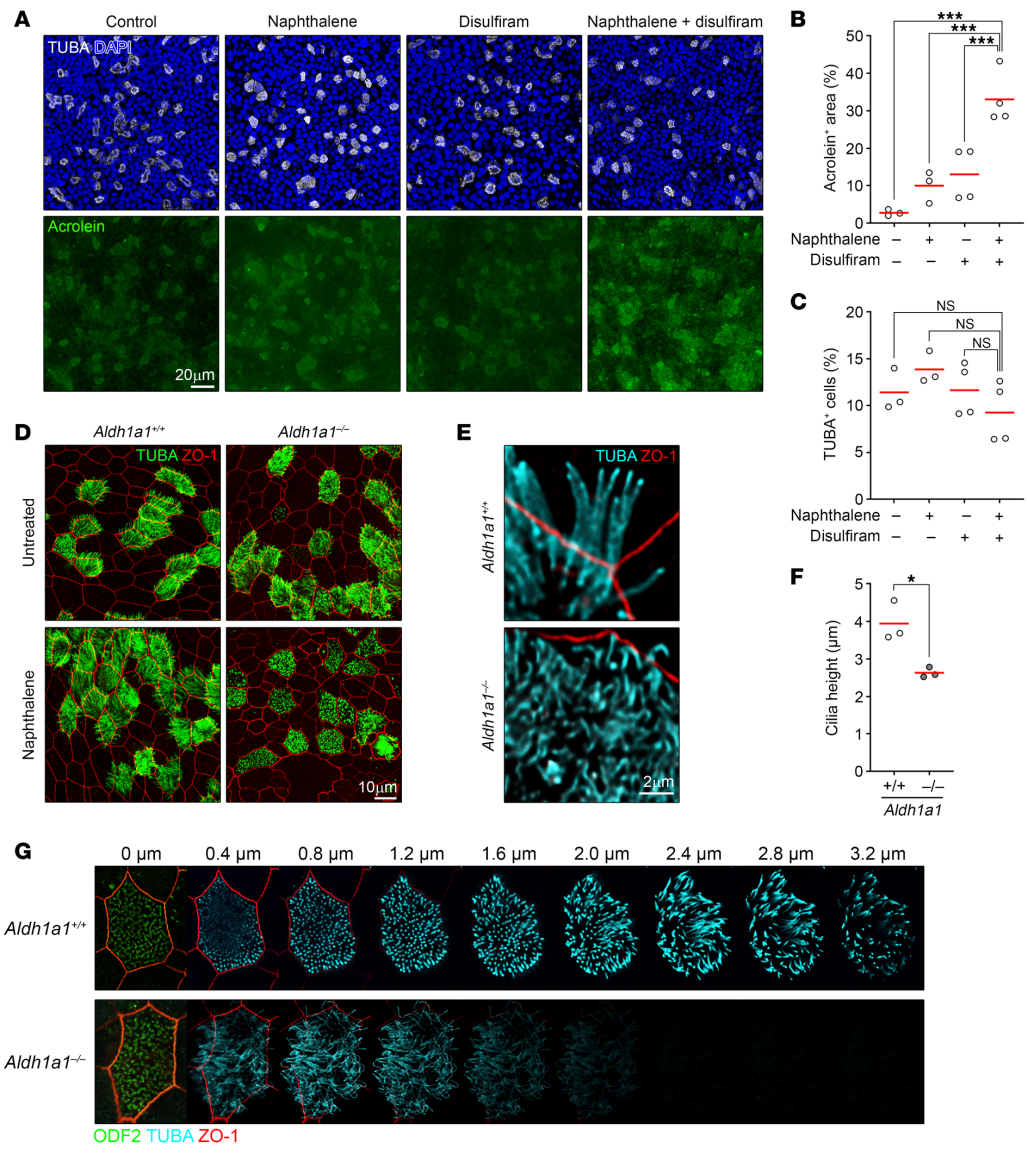
Aberrant cilia regeneration in ciliated cell culture. (**A**–**C**) Immunofluorescence staining of ALI culture with or without 10 μM naphthalene administration for 10 days in the presence or absence of 2 μM disulfiram was assessed for acrolein adduct, cilia (TUBA), and nuclei (DAPI). (**A**) Maximum intensity projections of *Z*-stack images are presented. Scale bar: 20 μm. (**B** and **C**) The proportion of acrolein positive area (**B**) and TUBA+ ciliated cells (**C**) are shown (*n* = 3–4). (**D**–**G**) *Aldh1a1*^+/+^ and *Aldh1a1*^–/–^ primary tracheal cells in ALI culture were stimulated with or without 10 μM naphthalene for 10 days, followed by culture in normal medium for an additional 4 days. (**D**) Representative images of immunofluorescence staining for cilia (TUBA) and tight junction (ZO-1). (**E**) A 3D reconstitution of ciliated cells with naphthalene administration. Scale bars: 10 μm (**D**), 2 μm (**E**). (**F**) Average cilia height after naphthalene administration is shown (*n* = 3). (**G**) Optical sectioning images of representative ciliated cells with naphthalene administration. Each *Z*-slice represents an increment of 0.4 μm, starting from ODF2^+^ basal bodies. Mean values are shown by red horizontal lines (**B**, **C**, and **F**). ***P < 0.01 and *P < 0.05 by 1-way ANOVA followed by post hoc Tukey’s test (**B** and **C**) or unpaired *t* test (**F**). Data represented at least 2 independent experiments with similar results (**A**, **D**, **E**, and **G**).

To further interrogate the aldehyde generation and aberrant cilia formation, we cultured primary mouse tracheal cells using the air–liquid interface (ALI) method. This method induced the differentiation of ciliated cells, identified by TUBA expression, which constituted approximately 10% of the total cells (Figure 4A). Although naphthalene exposure moderately increased acrolein adduct formation, the presence of disulfiram, a pan-inhibitor for ALDHs, further increased the accumulation of these adducts (Figure 4, A and B). Notably, despite exhibiting a moderate level of CYP2F2, ciliated cells showed higher accumulation of acrolein adducts compared with nonciliated cells, regardless of the exposure to oxidative stress (Figure 4A and Supplemental Figure 4). This suggests that ciliated cells are inherently more susceptible to reactive aldehyde species. Nonetheless, neither naphthalene nor disulfiram affected the proportion of the ciliated cell population (Figure 4C). The enhanced accumulation of aldehyde adducts with disulfiram supports our in vivo observation that ALDH1A1 plays a role in detoxifying reactive aldehydes, although disulfiram may affect additional targets beyond ALDHs (43, 44). To further clarify the role of ALDH1A1, we cultured *Aldh1a1*^+/+^ and *Aldh1a1*^–/–^ tracheal cells using the ALI method and subsequently exposed them to naphthalene. Whereas *Aldh1a1*^+/+^ ciliated cells showed no noticeable alteration of cilia morphology regardless of naphthalene exposure, we noticed that cilia of *Aldh1a1*^–/–^ ciliated cells, after naphthalene exposure, were not elongated like those of *Aldh1a1*^+/+^ ciliated cells and displayed a curled and twisted appearance (Figure 4, D and E). Consequently, cilia of *Aldh1a1*^–/–^ ciliated cells were not as upright as those of *Aldh1a1*^+/+^ cells after naphthalene exposure, while the structural arrangements of ODF2^+^ basal bodies, which anchor the individual cilia, appeared to be similar (Figure 4, F and G). Collectively, these results confirm that ALDH1A1 deficiency results in aberrant cilia formation in response to oxidative damage.

### ALDH1A1 deficiency leads to impaired MCC.

**Figure 5:**
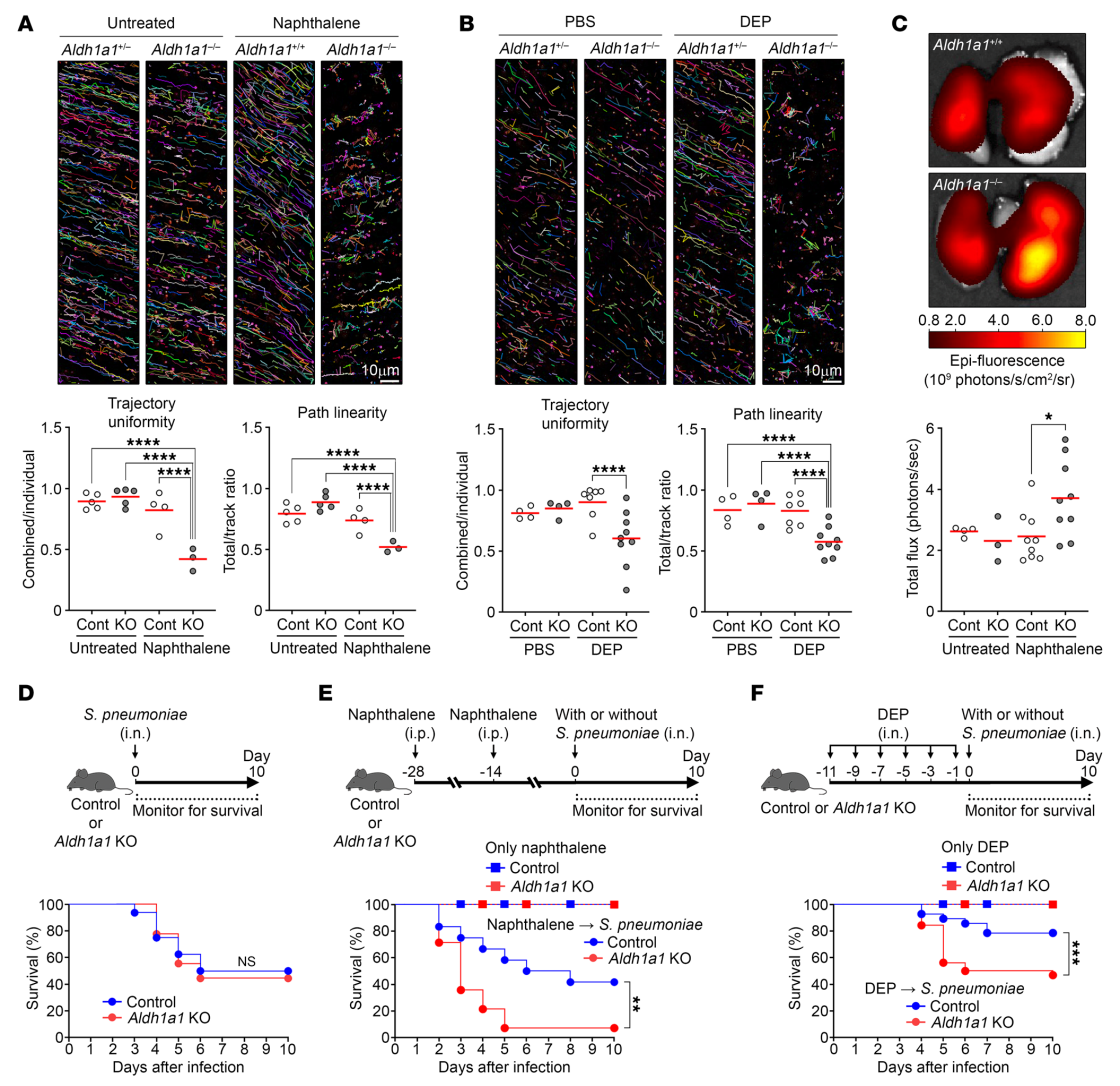
Impaired MCC in ALDH1A1-deficient mice. (**A**–**C**) Mice with indicated genotypes (control, *Aldh1a1*^+/+^ or *Aldh1a1*^+/–^; KO, *Aldh1a1*^–/–^) were intraperitoneally injected with 200 mg/kg naphthalene at 2-week intervals for a total of 2 times or were intranasally injected with 100 μg DEPs every other day for a total of 6 times. (**A** and **B**) Two weeks after the secondary naphthalene administration (**A**) or 3 days after the DEP administration (**B**), mucociliary transport in isolated tracheas was determined using live imaging of fluorescent beads. Each bead is assigned a specific color, and the motion of these beads over a 1-second time frame is demonstrated (upper). Trajectory uniformity calculated from 10 beads per recorded area (lower left) and path linearity of individual beads (lower right) are presented. Each dot represents one mouse, and the mean values are shown by red horizontal lines (**A**, *n* = 3–5; **B**, *n* = 4–9). Scale bars: 10 μm. (**C**) Two weeks after the second naphthalene injection, mice were intranasally injected with fluorescent microbeads, and bead accumulation in lung tissues was measured by IVIS at 24 hours after injection. Upper: Representative images of indicated genotypes with naphthalene exposure. Lower: Fluorescence intensity is shown. Each plot represents one mouse, and the mean values are shown by red horizontal lines (*n* = 3–9). (**B** and **F**) Mice with indicated genotypes were intranasally infected with 1 × 10^8^ CFU of *S*. *pneumoniae*. Schematic of experimental design (upper) and survival (lower) without any pre-exposure (**D**, *n* = 9–16), with naphthalene administration (**E**, *n* = 12–14), or with DEP administration (**F**, *n* = 28–29) are shown. ****P < 0.001, ***P < 0.01, **P < 0.02, *P < 0.05, and NS, not significant by 1-way ANOVA followed by post hoc Tukey’s test (**A**–**C**) or Kaplan-Meier survival analysis (**D**–**F**). Data represent at least 2 independent experiments with similar results (**A**–**C**).

In naphthalene-exposed *Aldh1a1*^–/–^ mice, most cilia exhibited atypical wave patterns, marked by reduced or absent beating frequency, suggesting impaired MCC function in these mice (Supplemental Video 1). To evaluate MCC functionality, we performed ex vivo live imaging to track the movement of fluorescent beads on the isolated tracheas (45). Under steady-state condition or with PBS administration, both control (*Aldh1a1*^+/+ or +/–^) and *Aldh1a1*^–/–^ tracheas displayed the uniform bead movement in the same observation area (trajectory uniformity) and consistent directionality of each bead (path linearity) (Figure 5, A and B, and Supplemental Videos 2 and 3). However, the bead movement on naphthalene- or DEP-exposed *Aldh1a1*^–/–^ tracheas became erratic and inconsistent, while that on control mice maintained uniformity and consistent flow. The MCC dysfunction in *Aldh1a1*^–/–^ mice remained for at least 6 weeks after naphthalene exposure (Supplemental Figure 5A). To further assess MCC function, we intranasally administrated 0.2 μm diameter fluorescent beads and measured their penetration into the respiratory tract 24 hours later. Lung imaging using an in vivo imaging system (IVIS) revealed increased bead infiltration into the lungs of naphthalene-exposed *Aldh1a1*^–/–^ mice (Figure 5C). These findings demonstrate that ALDH1A1 deficiency leads to impaired MCC function upon oxidative airway injury.

Considering the vital role of MCC in lower respiratory tract sterility, we next investigated whether ALDH1A1 deficiency affects susceptibility to respiratory infection. We intranasally infected mice with *S*. *pneumoniae*, the most common causative bacteria for community-acquired pneumonia. Both control and *Aldh1a1*^–/–^ mice exhibited similar susceptibility to *S*. *pneumoniae* in the absence of prior exposure (Figure 5D). However, after exposure to naphthalene or DEPs, *Aldh1a1*^–/–^ mice exhibited significantly higher mortality rates from *S*. *pneumoniae* infection compared with control mice (Figure 5, E and F). DEP exposure, but not naphthalene exposure, led to the recruitment of immune cells, including neutrophils and CD4 and CD8 T cells, into lungs, which likely contributed to enhanced resistance to *S*. *pneumoniae* infection (Supplemental Figure 5B). ALDH1A1 deficiency had minimal impact on leukocyte recruitment and the expression of chemokines and cytokines, suggesting that it does not substantially alter immune responses (Supplemental Figure 5, C and D). Although *S*. *pneumoniae* numbers in BALF show no significant difference between control and *Aldh1a1*^–/–^ mice, histological assessment revealed increased tissue damage and local *S*. *pneumoniae* burden in lungs of *Aldh1a1*^–/–^ mice (Supplemental Figure 5, E–G). Taken together, these findings suggest that ALDH1A1 deficiency increases pneumonia susceptibility due to the deeper penetration of bacteria into the respiratory tract, rather than affecting resistance immunity (46).

### Enhancing ALDH1A1 activity promotes regeneration of cilia and MCC.

**Figure 6:**
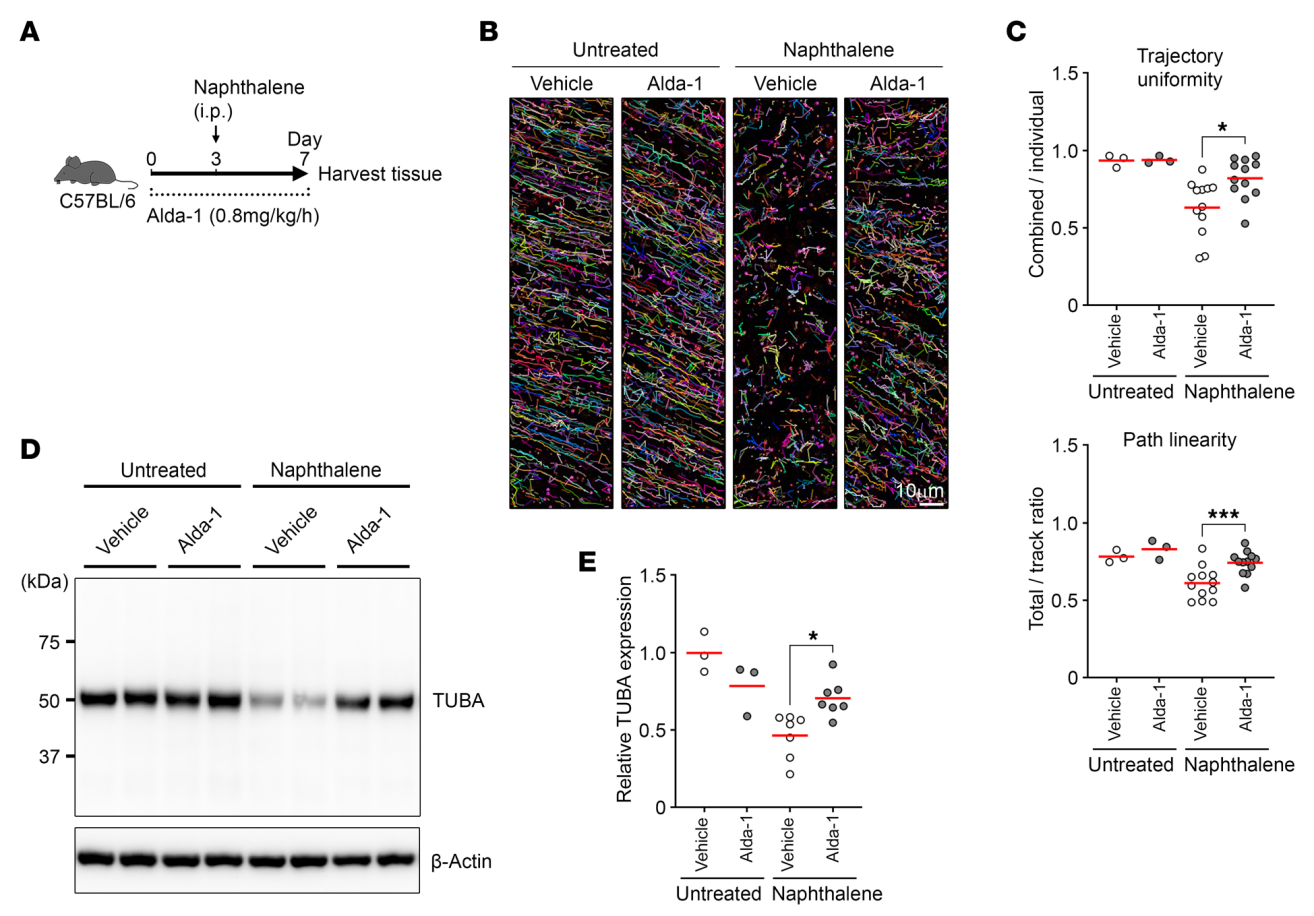
Enhancing ALDH1A1 activity promotes regeneration of cilia and MCC. WT mice were infused with vehicle or Alda-1 (0.8 mg/kg/h) via an implanted osmotic pump. Three days after infusion, mice were intraperitoneally injected with 200 mg/kg naphthalene, and trachea and lungs were harvested 4 days after naphthalene administration. (**A**) Schematic of experimental design. (**B** and **C**) Mucociliary transport in isolated tracheas was determined using live imaging of fluorescent beads. Each bead is assigned a specific color, and the motion of these beads over a 1-second time frame is demonstrated (**B**). Scale bar: 10 μm. Trajectory uniformity calculated from 10 beads per recorded area (upper) and path linearity of individual beads (lower) are presented (**C**, *n* = 3–12). (**D** and **E**) Lung lysates were analyzed by Western blot for TUBA and β-actin. Representative images of TUBA and β-actin (**D**). The average intensity of TUBA in vehicle-infused untreated samples was compared with the relative difference to that of other 3 groups (**E**, *n* = 3–7). Each point represents one mouse, and the mean values are shown by red horizontal lines (**C** and **E**). ***P < 0.01 and *P < 0.05 by 1-way ANOVA followed by post hoc Tukey’s test. Data represent at least 2 independent experiments with similar results (**B**).

People who survived acute respiratory distress syndrome, a major cause of mortality in patients with pneumonia, tend to exhibit elevated levels of ALDH proteins in their BALF when compared with nonsurvivors (47). Similarly, elevated ALDH1A1 levels have been reported in individuals exposed to emergency fire-induced smoke (48). Conversely, we found that patients with systemic respiratory diseases, including cystic fibrosis and chronic obstructive pulmonary disease (COPD), tended to show downregulation of *ALDH1A1* mRNA in ciliated cells (Supplemental Figure 6A). These observations imply the potential benefits of enhancing ALDH activity in the airway. Alda-1, a small compound originally identified as an activator of ALDH2, was recently shown to activate ALDH1A1 (49, 50). Alda-1 augments ALDH2 activity through binding near its Glu285 and Cys319 residues, which are also conserved in ALDH1A1 in both human and mouse (51). To investigate the therapeutic potential of enhancement of ALDH1A1 activity, we utilized an osmotic pump drug delivery system for the administration of Alda-1 in a sustained fashion (Figure 6A). While administration of Alda-1 did not affect the induction of ROS and lipid peroxidation in the airway epithelium, it significantly reduced the level of free MDA in BALF (Supplemental Figure 6, B–D). Furthermore, the administration of Alda-1 to C57BL/6 mice resulted in significantly better mucociliary transport activity compared with the vehicle control group after naphthalene exposure, while Alda-1 administration per se had no impact (Figure 6, B and C, and Supplemental Video 4). Additionally, the levels of TUBA in the lung tissues were higher in mice administrated with Alda-1 compared with vehicle control (Figure 6, D and E). These results indicate that enhancing ALDH1A1 activity can promote the regeneration of cilia and MCC upon oxidative airway injury.

## Discussion

The airway serves as the primary interface where inhaled PM_2.5_ first comes into contact. Therefore, defense strategies against noxious insults at this interface are crucial for preventing the adverse effects of PM_2.5_ exposure. In this study, we demonstrated that ALDH1A1-dependent aldehyde metabolism plays a crucial role in ciliary resilience upon PM_2.5_ exposure. Importantly, enhancement of ALDH1A1 activity improved the restoration of MCC function in response to oxidative airway injury. Targeting this mechanism may offer preventive strategies to mitigate the negative health consequences associated with PM_2.5_ exposure.

We assessed the detrimental effects of PM_2.5_ exposure using distinct experimental mouse models, including DEPs, PM, and naphthalene. According to calculations from previous studies (30, 52), the DEP dose employed in this study corresponds to a daily inhalation exposure of 855 μg/m^3^, a level often observed in regions of Asia, Africa, and the Middle East (53–55). Moreover, levels of PM_2.5_ exposure can substantially increase under certain circumstances. For example, while DEP concentrations on major streets and highways in the United States typically range from 20 to 25 μg/m^3^, pedestrians near vehicle exhaust emissions may experience much higher exposures, with averages exceeding 125 μg/m^3^ and peaks reaching 860 μg/m^3^ (56). Additionally, wildfires can expose populations to smoke plumes that travel hundreds of miles, raising PM_2.5_ levels exceeding 250 μg/m^3^ (57). These observations highlight that the DEP dose used in this study mirrors the relevant level of real-world human exposure.

We also employed naphthalene, a prominent PAH found in PM_2.5_, as a model to investigate oxidative airway injury resulting from PM_2.5_ exposure (58). Naphthalene has been extensively studied in the context of airway epithelial regeneration, and it primarily targets club cells, which express high levels of the naphthalene-metabolizing enzyme CYP2F2. Consistently, naphthalene exposure induced more severe exfoliation in club cells compared with ciliated cells (Figure 3F and Supplemental Figure 3C), suggesting that the initial redox stress from naphthalene metabolism occurs predominantly in club cells. Although the relative contributions of dying club cells and ciliated cells to generate reactive aldehyde species in naphthalene exposure remain unclear, our in vitro study demonstrated that ciliated cells accumulated increased levels of acrolein adducts compared with nonciliated cells (Figure 4A). This suggests that, regardless of the initial trigger of oxidative damage, ciliated cells are inherently more vulnerable to reactive aldehyde species. Their heightened susceptibility may be attributed to the unique structural features of ciliated cells, particularly their extended plasma membrane, which could enhance the generation of and/or exposure to reactive aldehydes.

The precise mechanism by which reactive aldehydes selectively impair cilia remains an intriguing area for further investigation. One potential explanation is that reactive aldehyde species generated near the ciliary plasma membrane interfere with the formation of cilia architecture. Indeed, acetylation of α-tubulin at Lys40, which is critical for the structural integrity of cilia, was impaired in *Aldh1a1*^–/–^ ciliated cells (Figure 3E). α,β-Unsaturated aldehyde species modify amino acids primarily at nucleophilic residues, including cysteine (thiol), lysine (ε-amino), and histidine (imidazole), through Michael addition (59). Among these aldehydes, 4-HNE and acrolein are particularly reactive toward proteins, forming stable covalent adducts with histidine, lysine, and cysteine residues, while MDA primarily targets lysine residues (60). It is therefore likely that Michael addition of α,β-unsaturated aldehyde species on Lys40 of α-tubulin compete with the acetylation on the same lysine residue, thereby destabilizing the microtubule architecture of cilia (61). However, it remains unclear whether α,β-unsaturated aldehydes also modify other critical components of cilia, thereby contributing to the impairment of MCC. Given the current limitations in precisely quantifying aldehyde-protein adducts at specific residues or cellular compartments, further studies are required to delineate the individual contributions of distinct α,β-unsaturated aldehydes to ciliary dysfunction.

ALDH1A1 is known for its involvement in RA synthesis, which is involved in epithelial cell differentiation and cilia formation (62). A recent study demonstrated that the disruption in RA signaling results in the expansion of hillock basal cells, which contribute to squamous metaplasia (42). However, in ALDH1A1-deficient mice, overall gene expression patterns, including RA-responsive genes and the hillock cell marker *Krt13*, remained largely unchanged (Supplemental Figure 3, D–G). Furthermore, in cultured *Aldh1a1*^–/–^ ciliated cells, abnormal cilia formation was observed even in the continuous presence of RA (Figure 4, D–G). These findings strongly suggest that impaired cilia regeneration in *Aldh1a1*^–/–^ mice is more likely a consequence of defective aldehyde detoxification rather than disrupted RA production.

Although this study illustrates the significance of aldehyde metabolism concerning PM_2.5_ in the context of pneumonia mortality, the impact of aldehyde metabolism remains to be investigated in other respiratory diseases, including asthma, COPD, and cystic fibrosis, which are also linked to PM_2.5_ exposure (63–65). Although we have not found specific SNPs of *ALDH1A1* associated with respiratory diseases in the GWAS Catalog (https://www.ebi.ac.uk/gwas/home), we found the *ALDH1A1* gene tended to be downregulated in ciliated cells from patients with systemic respiratory diseases, including cystic fibrosis and COPD (Supplemental Figure 6A). This suggests that, while genomic variants may not yet be linked, downregulation of *ALDH1A1* may lead to impaired MCC, contributing to the pathogenesis of these respiratory diseases. Further investigation is therefore required to assess the functional relevance of *ALDH1A1* downregulation in these diseases. Moreover, people living in modern society directly inhale harmful aldehydes from various sources, such as cigarette smoke/vapor and building materials that cause sick building syndrome (66–68). Further studies may also look to explore the significance of aldehyde metabolism against these endogenously and exogenously derived aldehydes in maintaining MCC homeostasis and in disease contexts.

## Methods

### Sex as a biological variable.

Our study examined both male and female mice, with similar findings observed in both sexes. However, in certain experiments as noted, only male mice were used to minimize variability in phenotype.

### Animals.

All mice were in the C57BL/6J background, and littermate controls were used for experiments when feasible. C57BL/6 mice were purchased from CLEA Japan and bred and housed under specific pathogen-free conditions. Mice deficient for *Aldh1a1* were generated by CRISPR/Cas9-mediated gene targeting in C57BL/6 zygotes using CRISPR RNA (5′-AGTTCTTAACCCTGCAACTG-3′) as targeting guide, and STOP sequences were inserted by electroporation. Mice were group-housed and fed standard chow at ambient temperature of 25°C with 50% humidity on average and a 12-hour light-dark cycle in individually ventilated cages.

### Reagents.

Naphthalene and disulfiram were purchased from FUJIFILM Wako Pure Chemical Corporation. DEPs (SRM2975; National Institute of Standards and Technology) and PM (SRM2786; National Institute of Standards and Technology) were purchased from Sigma-Aldrich. Alda-1 was purchased from MedChemExpress.

### Alda-1 in vivo administration.

Alda-1 (in 50% DMSO/50% PEG, at 0.8 mg/kg/h) or vehicle control (50% DMSO/50% PEG) was administered using an ALZET osmotic pump (DURECT Corp.) as described previously (69). Osmotic pumps were implanted 2 or 3 days before naphthalene or DEP exposure to minimize the impact of surgical procedure on the outcome of the experiments.

### Naphthalene exposure.

Eight- to nine-week-old mice (C57BL/6, *Aldh1a1*^–/–^, *Aldh1a1*^+/+^, and *Aldh1a1*^+/–^ littermates or age-matched pairs) received intraperitoneal injection of naphthalene (200 mg/kg). For trachea tissue live imaging, naphthalene was administered 4 h prior to imaging. For histology, immunofluorescence, electron microscopy, flow cytometry, mucociliary transport assay, RNA-seq, and pneumonia experiments, mice received the second naphthalene injection 2 weeks after the first injection. Two weeks after the second injection, mice were sacrificed or used for pneumonia experiments as described below.

For Alda-1 treatment, WT mice were divided into 4 groups: vehicle control without injury, Alda-1 without injury, vehicle control with naphthalene exposure, and Alda-1 with naphthalene exposure. Four days after intraperitoneal injection of naphthalene (200 mg/kg), mice were sacrificed and trachea and lung tissues were isolated for mucociliary transport assay and western blotting, respectively.

### DEP exposure.

DEP powder was suspended in PBS at 2 mg/mL (w/v), vortexed for 2 minutes, sonicated for 10 minutes in a cooled water bath, aliquoted, and stored at –80°C until use. Upon thawing for each experiment, aliquots were sonicated for 5 minutes immediately before use. Mice (C57BL/6, *Aldh1a1*^–/–^ versus WT or *Aldh1a1*^+/–^ littermates or age-matched pairs, aged 10–12 weeks) were lightly anesthetized with isoflurane and exposed to either 100 μg DEPs or PM in 50 μL of PBS or 50 μL PBS without DEPs or PM. For trachea tissue live imaging, DEPs or PM were administered twice at 2 and 16 h prior to imaging. For MDA measurement in BALF, DEPs were administrated 3 times per day for 2 consecutive days (6 times total). Alternatively, DEPs were administered 6 times every second day, and mice were sacrificed 24 h after the last exposure for quantitative PCR or 3 days after the last exposure for histology, immunofluorescence, flow cytometry, and mucociliary transport assay. Pneumonia experiments were performed 1 day after the sixth exposure as described below.

### MDA measurement.

After DEP or PBS administration, the trachea was exposed under deep anesthesia. BALF was collected in 1 mL of PBS using a 22G catheter and centrifuged at 1,000*g* at 4°C for 5 minutes, and the supernatant was collected for assay. The concentration of free MDA was quantified utilizing an aromatic hydrazine-based method, employing the Colorimetric Lipid Peroxidation (MDA) Assay Kit (Abcam).

### Histology and immunofluorescence staining of lung tissue sections.

Mice were sacrificed using CO_2_ inhalation. Isolated lung tissues were fixed with 4% paraformaldehyde (PFA) overnight at 4°C and embedded in paraffin. Tissue sections (6 μm thickness) were prepared using a microtome (SLEE medical) and mounted onto adhesive glass slides (Matsunami Glass). Rehydration was performed with xylene, followed by a standard ethanol dilution series. For histology, sections were stained with H&E solutions (FUJIFILM Wako). For immunostaining, antigen retrieval was performed at 98°C for 45 minutes using an ImmunoSaver device (FUJIFILM Wako). Sections were permeabilized with 0.1% Triton X-100 for 10 minutes and then blocked with Blocking One Histo buffer (Nacalai Tesque) at room temperature for 1 hour. After blocking, sections were incubated with primary antibodies diluted in Can Get Signal immunoreaction enhancer solution (Toyobo) overnight at 4°C: ALDH1A1 (Cell Signaling Technology; D4R9V), 4-HNE (JaICA; HNEJ-2), CC10 (Abcam; EPR19846), CYP2F2 (Santa Cruz Biotechnology; F-9), and *S*. *pneumoniae* (Abcam; ab20429). After incubation with fluorescently conjugated secondary antibodies, nuclear staining with DAPI (100 nM in PBS, 5 minutes) was performed after the final antibody application. After DAPI staining, slides were washed 4 times in PBS and sealed in mounting medium (Prolong Diamond Antifade Mountant; Thermo Fisher Scientific) for microscopy observation.

### ROS, lipid peroxide, and acrolein labeling, and plasma membrane staining of isolated trachea tissues for live imaging.

Mice were euthanized by CO_2_ inhalation and tracheas were dissected. For ROS and lipid peroxide labeling, isolated trachea tissues were incubated with LipiRADICAL Green (2.5 μM; Funakoshi), CellROX Deep Red (10 μM; Thermo Fisher Scientific), and CellTracker Red CMTPX (1:1,000 dilution; Thermo Fisher Scientific) in DMEM/F12 without phenol red (Thermo Fisher Scientific) for 30 minutes at 37°C. For acrolein labeling, trachea tissues were incubated with AcroleinRED (10 μM; Funakoshi) and CellMask Plasma Membrane Deep Red (1:1,000 dilution; Thermo Fisher Scientific) for 20 minutes at 37°C. For time-lapse recording of cilia movement, trachea tissues were incubated with CellMask Plasma Membrane Orange (Thermo Fisher Scientific) at 1:1,000 dilution for 30 minutes at room temperature. After labeling, tissues were rinsed with DMEM/F12 twice and mounted using medical adhesive (Daiichi Sankyo) onto glass slides with 0.4 mm high ridges, covered with DMEM/F12, and sealed with cover glasses for microscopy observation.

### Airway epithelial injury model in vitro.

C57BL/6 male mice (6–8 weeks) were euthanized by CO_2_ inhalation, and ALI cultures of mouse airway epithelial cells were prepared from trachea and main bronchi without expansion, as described previously (70, 71). Briefly, after enzymatic digestion and fibroblast deprivation (70, 71), collected nonadherent cells were resuspended in MTEC proliferation medium: DMEM/F12 supplemented with 5% (v/v) FBS, Insulin-Transferrin-Selenium (Thermo Fisher Scientific), 1.5 mM l-glutamine, 0.1 μg/mL cholera toxin, 0.025 μg/mL murine EGF (PeproTech Inc.), 0.03 mg/mL bovine pituitary extract (Thermo Fisher Scientific), Y-27632 (Cayman Chemical), 0.05 μM RA (Sigma), and antibiotics (Penicillin-Streptomycin-Amphotericin B mixture; Lonza). The cells were then seeded onto a 6.5 mm Transwell 0.4 μm pore polyester membrane insert (Corning) at 8 × 10^4^ cells/cm^2^ and incubated at 37°C with 5% CO_2_. At 100% confluence, differentiation was induced by removing apical media and replacing the basal media with differentiation medium: DMEM/F12 supplemented with 0.1% (w/v) Bovine Albumin Fraction V (Thermo Fisher Scientific), Insulin-Transferrin-Selenium (Thermo Fisher Scientific), 1.5 mM l-glutamine, 0.025 μg/mL cholera toxin, 0.005 μg/mL murine EGF (PeproTech Inc.), 0.03 mg/mL Bovine Pituitary Extract (Thermo Fisher Scientific), 0.1 μM RA (Sigma), and antibiotics (Penicillin-Streptomycin-Amphotericin B mixture; Lonza). For naphthalene-induced injury, naphthalene was applied in the basal differentiation medium at 10 μM for 10 days from differentiation day 7. After naphthalene exposure, cells were incubated in normal differentiation medium for 4 days, in the presence or absence of pan-ALDH inhibitor disulfiram (2 μM), depending on the experimental design. Ethanol and DMSO were used as carrier controls for naphthalene and disulfiram, respectively.

After exposure, cells on the membrane insert were fixed with 4% (w/v) PFA in PBS for 15 minutes at room temperature. After fixation, membranes were removed from the inserts and placed on a glass slide for immunostaining. After washing with PBS containing 0.05% (v/v) Triton X-100 (PBS-T), cells on the membrane were blocked with 5% (w/v) BSA in PBS-T for 1 hour; incubated with a combination of primary antibodies against acrolein (Abcam; 10A10), ALDH1A1 (Cell Signaling Technology; D4R9V), ZO-1 (Invitrogen; ZO1-1A12), ODF2 (Abcam; ab43840), or TUBA (Santa Cruz Biotechnology; 6-11B-1) in PBS-T containing 2% (w/v) BSA overnight at 4°C; and incubated with fluorescently conjugated secondary antibodies in PBS-T containing 2 % (w/v) BSA for 1 hour. After nuclear staining with DAPI in PBS-T for 5 minutes, membranes were washed with PBS-T and sealed with a drop of mounting medium (Prolong Diamond Antifade Mountant; Thermo Fisher Scientific) for microscopy observation.

### Microscopy imaging and analyses.

H&E images and some of the immunofluorescence images were captured using a BZ-X800 microscope (Keyence Corp.). Confocal imaging was performed using a STELLARIS 5 WLL confocal microscope (Leica Microsystems) equipped with LAS X software.

For trachea tissue live imaging, *Z*-stack images were captured using the confocal microscope, and a confocal image with maximal fluorescence intensity was selected for each analysis. CellROX positive and LipiRADICAL Green positive cells per 200 × 200 (40,000) μm^2^ were counted using ImageJ (NIH). For quantitative analyses of acrolein adducts in ALI culture (in vitro), *Z*-stack images were captured using the confocal microscope every 1 μm for a total of 34 sections, and merged gray scale images were analyzed using ImageJ software. For cilia height measurement, ALI culture samples from *Aldh1a1*^+/+^ and *Aldh1a1*^–/–^ with or without naphthalene exposure were immunolabeled for TUBA and ZO-1, and fluorescence images were captured using the confocal microscope, as described above. The 3D reconstitutions of *Z*-stack images were used for cilia height measurement. For each sample, 2 images (96.88 × 96.88 μm^2^, each containing 20–30 ciliated cells) were captured, and cilia height was determined as the distance from ZO-1 to the top of the TUBA signal in each ciliated cell. Approximately 50 ciliated cells per sample were analyzed. Image analysis was performed using ImageJ.

For quantifying the percentage of ciliated epithelial surfaces in lung tissue sections, immunofluorescence images were captured using the BZ-X800 microscope from the top to the bottom of 6 μm sections every 0.6 μm and merged, and the total epithelial length and ciliated surface length of tissue samples were manually measured using the line tool of ImageJ.

For detailed morphological observation of cilia, super-resolution microscopy was performed using a Nikon AX R Confocal Microscope System with a Spatial Array Confocal detector equipped with ×100 objective lens (PLAN APO λD ×100 /1.45 oil) and NIS-Elements image acquisition software. Imaging of fixed samples was performed using Galvano mode. For recording cilia movement, time-lapse imaging was performed using resonant mode at 29.3 fps.

### Scanning electron microscopy.

Tissue samples were fixed by perfusion with 2% formaldehyde and 2.5% glutaraldehyde in 0.1 M phosphate buffer (pH 7.4), sliced into 2 mm pieces, and immersed in the same fixation buffer. After washing, the specimens ware postfixed with 1% osmium tetroxide in 0.1 M phosphate buffer (pH 7.4) containing 1% potassium ferrocyanide and conductive stained with 1% tannic acid solution and 1% osmium tetroxide solution. The specimens were dehydrated in a graded series of ethanol, substituted with 100% ethanol, dried by the critical-point drying method, and coated with osmium tetroxide by the vacuum deposition method. Electron micrographs were captured with an S-4800 field emission scanning electron microscope (Hitachi High-Technologies Corp.).

### Mucociliary transport assay.

Mucociliary transport was analyzed using fluorescent beads (Fluoresbrite, 0.5 μm; Polysciences) as described previously (45). Briefly, after removing unnecessary surrounding tissues, an isolated trachea was opened from the dorsal side. With luminal surface facing upward, the tissue was placed in a rectangular space surrounded by 0.4 mm^2^ high vinyl ridges on a glass slide. The tissue was secured using medical adhesive (Daiichi Sankyo) as described above. Immediately after applying a drop of prewarmed fluorescent beads (1:500 dilution in DMEM/F12; 37°C) onto the luminal surface, a cover glass was placed on top for microscopy observation. Time-lapse images were recorded at 136 ms intervals using the STELLARIS 5 WLL confocal microscope with ×20 objective lens. Beads were tracked and analyzed using the TrackMate plug-in for Fiji (72–74). Traveling linearity and directional uniformity were calculated by analyzing 10 individual beads per record. Two recordings were performed for each trachea. In each record, 300–600 beads were detected within the area (258.33 × 64.20 μm), and 10 beads with recording of more than 6 continuous frames (0.816 seconds) were randomly selected for calculations. For individual beads, displacement α was defined as the direct distance from initial position (frame *t* = 0, coordinate: *x*_t = 0_, *y*_t = 0_) to the endpoint (frame *t* = 6, coordinate: *x*_t = 6_, *y*_t = 6_), and total track distance β was defined as the total traveling distance within the same time frame (from *t* = 0 to *t* = 6). Traveling linearity was calculated by dividing α by β.

 (Equation 1)
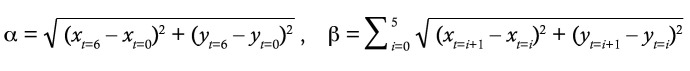


Traveling linearity = α/β

For calculating directional uniformity, the individual trajectory vector ϕ was determined as displacement from frame 0 to frame 6, and the average trajectory vector Φ was defined as the average of 10 individual trajectory vectors within the same record. Directional uniformity was calculated by dividing the length of Φ (displacement as a group) by the average size of 10 individual ϕ vectors (individual displacement).

 (Equation 2)
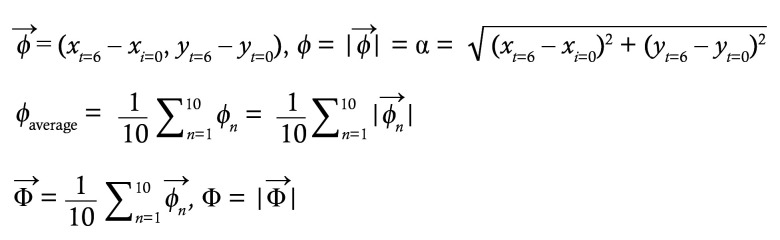


Directional uniformity = Φ/ ϕ_average_

### In vivo imaging.

Mice were anesthetized using an anesthetic combination (medetomidine, midazolam, and butorphanol), and fluorescent carboxylate–modified microspheres with a diameter of 0.2 μm (1:100 dilution; Invitrogen; F8807) were administered intranasally. At 1, 6, and 24 hours after administration, in vivo imaging was performed using IVIS Lumina III version 4.7 (PerkinsElmer). Settings for imaging were as follows: lamp level, low; excitation, 680 nm; emission, 790 nm; epifluorescence; binning, medium; field of view, A; F-stop = 2; and acquisition time = 1 s. Total flux (photons/s) was measured within equally sized rectangular regions of interest using Living Image software (PerkinElmer).

### Flow cytometry.

After removal of blood cells by cardiac perfusion with 5 mL of ice-cold PBS via the right ventricle and instillation of dissociation buffer (HBSS containing 0.2 U/mL Liberase and 20 μg/mL DNase I, both from Roche) via a trachea catheter, lung lobes were isolated. Minced lung tissues were digested in dissociation buffer at 37°C for 30 minutes. The reaction was stopped by adding an excess volume of buffer containing 10% (v/v) FBS, and the mixtures were filtered through a 70 μm filter to remove undigested parts and debris. After blocking with anti-mouse CD16/CD32 (BioLegend: 93), leukocytes were labeled using combinations of fluorophore-conjugated antibodies: anti-CD45 (BioLegend; 30-F11), anti-CD11b (BioLegend; M1/70), anti-Ly6G (BioLegend; 1A8), Ly6C (BioLegend; HK1.4), anti-CD11c (BioLegend; N418), anti-SiglecF (BD Biosciences; E50-2440), anti-CD4 (BioLegend; GK1.5), and anti-CD8a (BioLegend; 53-6.7).

For labeling epithelial populations, digested tissues were cleaned using a Debris Removal kit (Miltenyi) and then blocked with anti-mouse CD16/CD32. Cell surface markers were labeled using anti-CD45, anti-CD31(BioLegend; 390), and anti-CD326 (BioLegend; G8.8). After labeling dead cells using Zombie Aqua (BioLegend), cells were fixed and permeabilized using the Cytofix/Cytoperm Fixation/Permeabilization kit (BD Biosciences). Intracellular airway epithelial marker proteins were labeled using anti-CYP2F2 (Santa Cruz Biotechnology; F-9) and anti-TUBA (Cell Signaling Technology; D20G3). After staining, cell suspensions were filtered through a 70 μm strainer. Flow cytometry was performed on an LSRFortessa system (BD Biosciences).

### Real-time RT-PCR.

Total RNA extraction was performed using an RNeasy Mini Kit (Qiagen) for trachea tissues and Trizol Reagent (Thermo Fisher Scientific) for the other tissues. cDNA was prepared using PrimeScript RT Master Mix (Takara Bio Inc.) containing random hexamer and oligo-dT primer. Alternatively, SMART MMLV Reverse Transcriptase (Clontech) with oligo-dT primer was used. Real-time PCR was performed using KAPA SYBR Fast (KAPA Biosystems) and the CFX Connect Real-Time System (Bio-Rad). The primers used for the mouse genes are as follows: *Gapdh*, 5′-GTTGTCTCCTGCGACTTCAAC-3′ and 5′-CCAGGGTTTCTTACTCCTTGG-3′; *Rpl13a*, 5′-GGCTGAAGCCTACCAGAAAGT-3′ and 5′-TCTTTTCTGCCTGTTTCCGTA-3′; *β-actin*, 5′-TGTTACCAACTGGGACGACA-3′ and 5′-GGGGTGTTGAAGGTCTCAAA-3′; *Aldh1a1*, 5′-GGCTTAATCCAACAGATTCATTCACCT-3′ and 5′-ACACCTGGGGAACAGAGCA-3′; *Aldh1a2*, 5′-CACAGGAGAGCAAGTGTGTGA-3′ and 5′-TAGTTGCAAGAGTTGCCCTGT-3′; *Aldh1a3*, 5′-AAACCCACGGTCTTCTCAGAT-3′ and 5′-CTTTGTCCAGGTTTTTGGTGA-3′; *Aldh1a7*, 5′-AGCTTAATCTGGCAGAATCAGAGTCT-3′ and 5′-TCAGAGGAATAACCCCGAGGAAT-3′; *Aldh2*, 5′-TTTATGAACAGTGGCCAGACC -3′ and 5′-TCGTTGATGATCCTCCCATAG-3′; *Aldh3a1*, 5′-GATCCTAACTCCAAGGTGATGC-3′ and 5′-ACCCGTTTGATGAGCTTATTGT-3′; *Aldh3a2*, 5′-GATCCTAACTCCAAGGTGATGC-3′ and 5′-ACCCGTTTGATGAGCTTATTGT-3′; *Aldh3b1*, 5′-GAAGCATTTCAAGCGACTCC-3′ and 5′-CAGGCTTCTCACAGTCACCA-3′; *Aldh3b2*, 5′-GCAACGATGGCTTCCTCTAC-3′ and 5′-AGCCTATGGCCCAGCTTATC-3′; *Aldh3b3*, 5′-AGCGCTTTATGCCTATTCCA-3′ and 5′-ACGGAGGCCATTAAGCTTCT-3′; *Aldh4a1*, 5′-TGGAAGCACACCTCCTCTCT-3′ and 5′-AAGGGCGACAACTGGTACTG-3′; *Aldh5a1*, 5′-TTACTGGCTCAACAGCAACG-3′ and 5′-TGTTTGAGCAAACGCAAGTC-3′; *Aldh6a1*, 5′-ATCCTCGTAGGGGAGGCTAA-3′ and 5′-TTAATTCTTCGCCCATCCAG-3′; *Aldh7a1*, 5′-GGAAGGAATAGGCGAGGTTC-3′ and 5′-AGTGATGATTCCCACCAAGC-3′; *Aldh8a1*, 5′-GCAAAGCACATTTGGAGAAAG-3′ and 5′-AGCGGGACTCATCCTTAATGT-3′; *Aldh9a1*, 5′-GGCCAGTTTCTGTGTCATCAT-3′ and 5′-CCCTTCACAGCATTCTCCATA-3′; *Aldh16a1*, 5′-CTTCTCCTTTCCGCACAGTC-3′ and 5′-CCATGAGCATTGATCCACAC-3′; *Aldh18a1*, 5′-ATGGTTACCGCTTTGGACTG-3′ and 5′-CTTCCATGCTCGGAGAAGTC-3′; *Txnrd1*, 5′-CAGTTCGTCCCAACGAAAAT-3′ and 5′-GCACATTGGTCTGCTCTTCA-3′; *Hmox1*, 5′-TGCTCGAATGAACACTCTGG-3′ and 5′-TCTCTGCAGGGGCAGTATCT-3′; *Foxj1*, 5′-CAGACCCCACCTGGCAGA-3′ and 5′-TGAAGGCCCCACTGAGCA-3′; *Muc5ac*: 5′-AGTTGCCAGTGTCTACAGCC-3′ and 5′-CTGGAAGTCATCAGCCTGCA-3′; *Scgb1a1*, 5′-ACAATCACTGTGGTCATGCTGT-3′ and 5′-AGGGTATCCACCAGTCTCTTCA-3′; *Trp63*: 5′-GTCAGCCACCTGGACGTATT-3′ and 5′-CTCATTGAACTCACGGCTCA-3′; *Krt13*: 5′-AACAAGGCTGGAACAGGAGA-3′ and 5′-CACATCCTGCAGTCCTCTCA-3′; *Cxcl1*, 5′-GCTGGGATTCACCTCAAGAA-3′ and 5′-TCTCCGTTACTTGGGGACAC-3′; *Ccl2*, 5′-AGGTCCCTGTCATGCTTCTG-3′ and 5′-TCTGGACCCATTCCTTCTTG-3′; *Csf2*, 5′-GGCCTTGGAAGCATGTAGAG-3′ and 5′-CCGTAGACCCTGCTCGAATA-3′; *Il1b*, 5′-TGTGGCAGCTACCTGTGTCT-3′ and 5′-TGTTCATCTCGGAGCCTGTA-3′; *Il6*, 5′-AAGCCAGAGTCCTTCAGAGAGATA-3′ and 5′-CAGGGGTGGTTATTGCATCT-3′; *Il17a*, 5′-TCCAGAAGGCCCTCAGACTA-3′ and 5′-AGCATCTTCTCGACCCTGAA-3′.

### RNA-seq.

Lung tissues were isolated from 2 mice per group (*Aldh1a1*^+/+^ and *Aldh1a1*^–/–^) and digested as described above for RNA extraction and purification. RNA-seq sample preparation was performed using an RNeasy Mini Kit (Qiagen). Sequencing libraries were constructed through library preparation following the recommended protocol for the TruSeq stranded mRNA Library Prep kit (Illumina). Fragment size of the libraries was confirmed with a LabChip DNA High Sensitivity Reagent Kit (PerkinElmer). Libraries were sequenced on a NovaSeq 600 (Illumina) in the 101-base single-read mode. Among the known RA-responsive 532 genes (75, 76), 153 genes with more than 100 reads were selected, as shown in Supplemental Table 1, and plotted against all genes (log_2_ [fold change] [*x* axis] against average fragments per kilobase million [*y* axis]). Pathway analysis was performed using integrated differential expression and pathway analysis (iDEP2.0, GAGE) using the Gene Ontology database for biological processes and TF.Target.RegNetwork for RAR target genes, respectively.

### Open-source data exploration.

RNA-seq data for human *ALDH* family genes were downloaded via the ENCODE Expression Atlas on July 10, 2023 (77, 78). Mouse and human lung single-cell data were sourced from the LungMAP Consortium (U01HL122642) (LungMAP IDs: LMEX0000004396 for human and LMEX0000004397 for mouse) using the human or mouse ShinyCell browser and downloaded from www.lungmap.net (LungMAP Data Coordinating Center; 1U01HL122638) on September 2, 2023. *ALDH1A1* expression levels in ciliated cells from healthy donors and patients were analyzed using a publicly available dataset (https://cellxgene.cziscience.com/collections/6f6d381a-7701-4781-935c-db10d30de293) using BBrowser X (BioTuring Inc., study 846a6f259f9d4d85b07789b03eb4e4aa) (79).

### Bacterial pneumonia.

For the bacterial pneumonia model, *S*. *pneumoniae* strain TIGR4 was used (80). Male C57BL/6 mice aged 10–13 weeks, pre-exposed to either naphthalene or DEPs, were used for bacterial pneumonia experiments. Mice were anesthetized by intraperitoneal injection of anesthetic mixtures (medetomidine, midazolam, and butorphanol) before infection and intranasally instilled with 1–2 × 10^8^ CFU of TIGR4 in 20 μL PBS. Bacterial culture, instillation, and CFU determination were performed as described previously (80).

### Western blot.

For ALDH1A1 detection, lung lysates were prepared using SDS sample buffer (62.5 mM Tris-HCl, pH 6.8, 2% SDS, 10% glycerol, 5% 2-mercaptoethanol, and 0.001% bromophenol blue), separated by electrophoresis on 4%–15% polyacrylamide gel, and transferred onto a PVDF membrane. The membrane was incubated with antibodies against ALDH1A1 or β-actin in EveryBlot (Bio-Rad), followed by incubation with antibodies against rabbit IgG conjugated with HRP. For TUBA detection, lung lysates were prepared using RIPA buffer, and protein concentrations were determined by the bicinchoninic acid method for loading normalization. Lysates were heat denatured in SDS sample buffer, separated on 10% polyacrylamide, and transferred onto PVDF membrane. The membranes were incubated with anti-TUBA (Cell Signaling Technology; D20G3, 1:1,000) followed by HRP-conjugated anti-rabbit IgG, or with HRP-conjugated anti–β-actin (13E5, 1:1,000) in Can Get Signal reagent. The peroxidase activity was detected by ImmunoStar Zeta (FUJIFILM Wako).

### Statistics.

All experiments were conducted at least twice, and statistical analyses (1-way ANOVA followed by post hoc tests or 2-tailedStudent’s *t* test) were performed using GraphPad Prism 9. Data points and mean values are presented unless otherwise noted. Mouse survival curves were compared with the log-rank test. *P* < 0.05 was considered statistically significant. Samples sizes are detailed in figure legends.

### Study approval.

All procedures were approved by the IACUC of The University of Osaka.

### Data availability.

Sequencing data are available from the National Center for Biotechnology Information Gene Expression Omnibus (GSE267105, GSE287365, and GSE296445). All data are available in the main text or the supplemental materials.

## Author contributions

NS and YO conceived the project idea, designed experiments, and wrote the manuscript. NS carried out most of the experiments, with assistance from HK and YO. TY contributed to the initial implementation of the study. JS contributed expertise and helped acquire funding. MY and SK provided *S*. *pneumoniae*. All authors participated in editing the manuscript.

## Supplementary Material

Supplemental data

Unedited blot and gel images

Supplemental table 1

Supplemental video 1

Supplemental video 2

Supplemental video 3

Supplemental video 4

Supporting data values

## Version 1 05/23/2025

In-Press Preview

## Version 2 07/15/2025

Electronic publication
