# Supplementary material for: Aldehyde metabolism governs resilience of mucociliary clearance to air pollution exposure
Source: J Clin Invest. 2025 May 15;135(14):e191276. doi: 10.1172/JCI191276 (PMC12259252; doi:10.1172/JCI191276)
Supplement: Supplemental data [file jci-135-191276-s103.pdf]

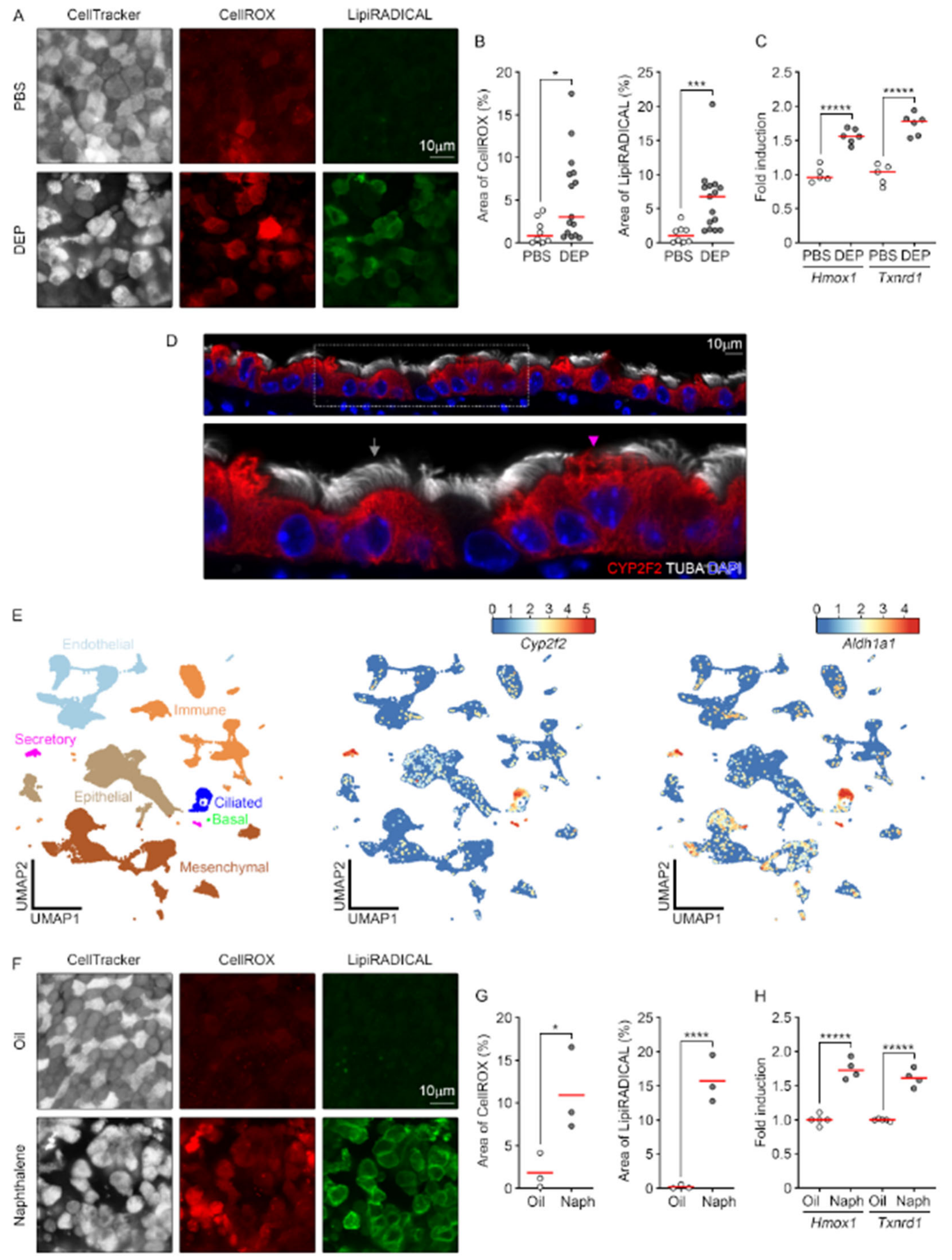

Supplemental Figure 1: $PM_{2.5}$ induces reactive aldehyde species in the airway

A-C WT mice were intranasally administered with 100 μg DEP at two hours and sixteen hours prior to dissection. **A** Luminal surface of isolated tracheas was determined for cell

shape (CellTracker), oxidative stress (CellROX), and lipid peroxidation (LipiRADICAL) and representative live images are shown. **B** The proportions of the area that were positive for CellROX (left) and LipiRADICAL (right) in (A) were determined (n=8-15). **C** Quantitative PCR for oxidative stress-responsive genes (*Hmox1* and *Txnrd1*) in lung at twenty-four hours post DEP injection (n=5-6). **D** Bronchial epithelium of WT mice were stained for CYP2F2, Cilia (TUBA), and nuclei (DAPI), and representative immunofluorescence images are shown. Lower: Enlarged images of gated region. Gray arrow: ciliated cell, pink arrow head: secretory cell. **e** UMAP visualization of color-coded mouse lung cell populations (left), *Cyp2f2* mRNA (middle), and *Aldh1a1* mRNA (right) projection with highest normalized expression level are shown. **F-H** WT mice were intraperitoneally injected with oil or 200 mg/kg naphthalene. **F** Four hours post naphthalene injection, the luminal surface of isolated tracheas was determined for cell shape (CellTracker), oxidative stress (CellROX), and lipid peroxidation (LipiRADICAL) and representative live images are shown. **G** The proportions of the area that were positive for CellROX (left) and LipiRADICAL (right) in (E) were determined (n=3). **H** Quantitative PCR for oxidative stress-responsive genes (*Hmox1* and *Txnrd1*) in lung at twenty-four hours post oil or naphthalene injection (n=4-5). Each point represents one mouse and the mean values are shown by red horizontal lines (B, C, G, and H). *****P<0.0001, ****P<0.001, ***P<0.01, and *P<0.05 by unpaired t-test. Data are represented by at least three independent experiments with similar results (A, D, and F).

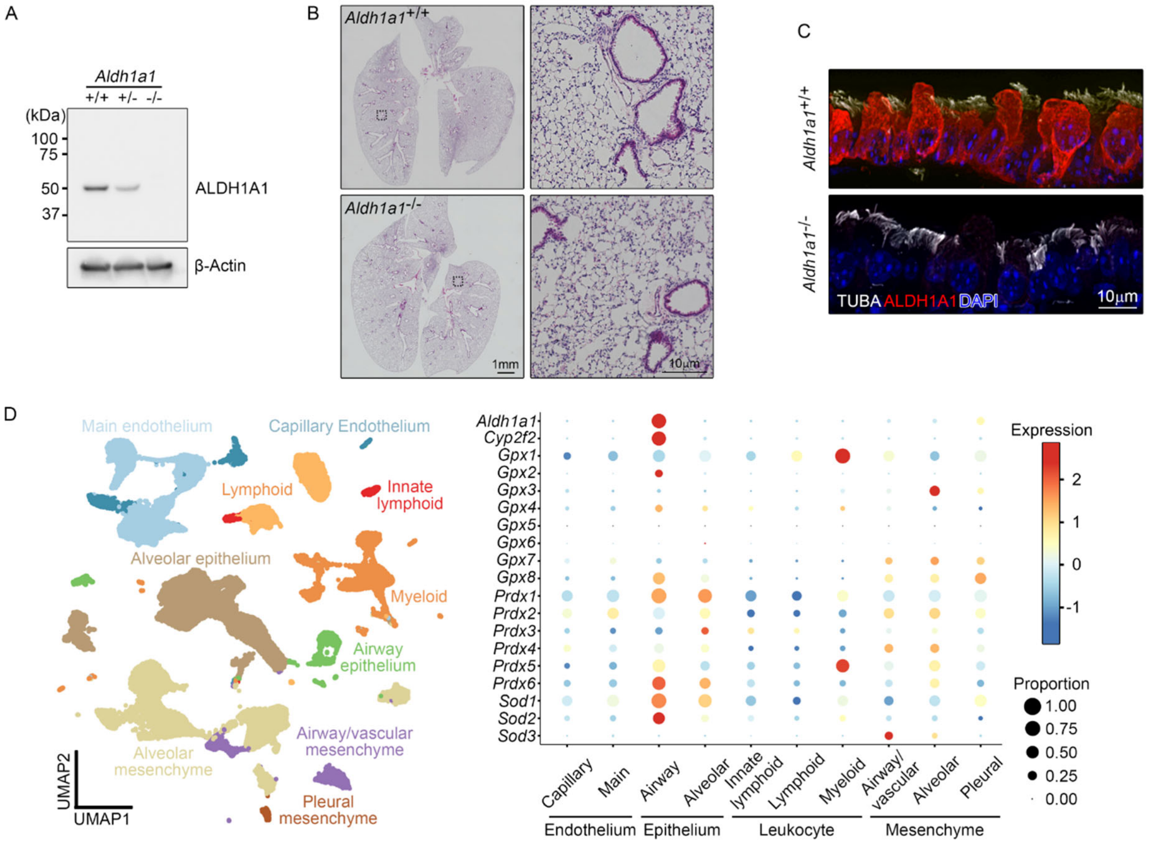

Supplemental Figure 2: Essential role of ALDH1A1 in detoxifying reactive aldehyde species in airway

A Lung lysates of indicated genotypes were analyzed by Western Blot for ALDH1A1 and β-Actin. **B** Hematoxylin and eosin (H&E) staining of lungs of indicated genotypes. Gated area in left are shown at a higher magnification in right. **C** Bronchial epithelium of indicated genotypes were stained for Cilia (TUBA), ALDH1A1, and nuclei (DAPI), and representative immunofluorescence images are shown. **D** UMAP visualization of color-coded annotation of mouse lung cell populations in scRNA-seq (left). Dot plots demonstrating scaled gene expression and percentage of cells expressing *Aldh1a1*, *Cyp2f2*, and anti-oxidant genes in indicated cell populations. Data are represented by at least three independent experiments with similar results (A-C).

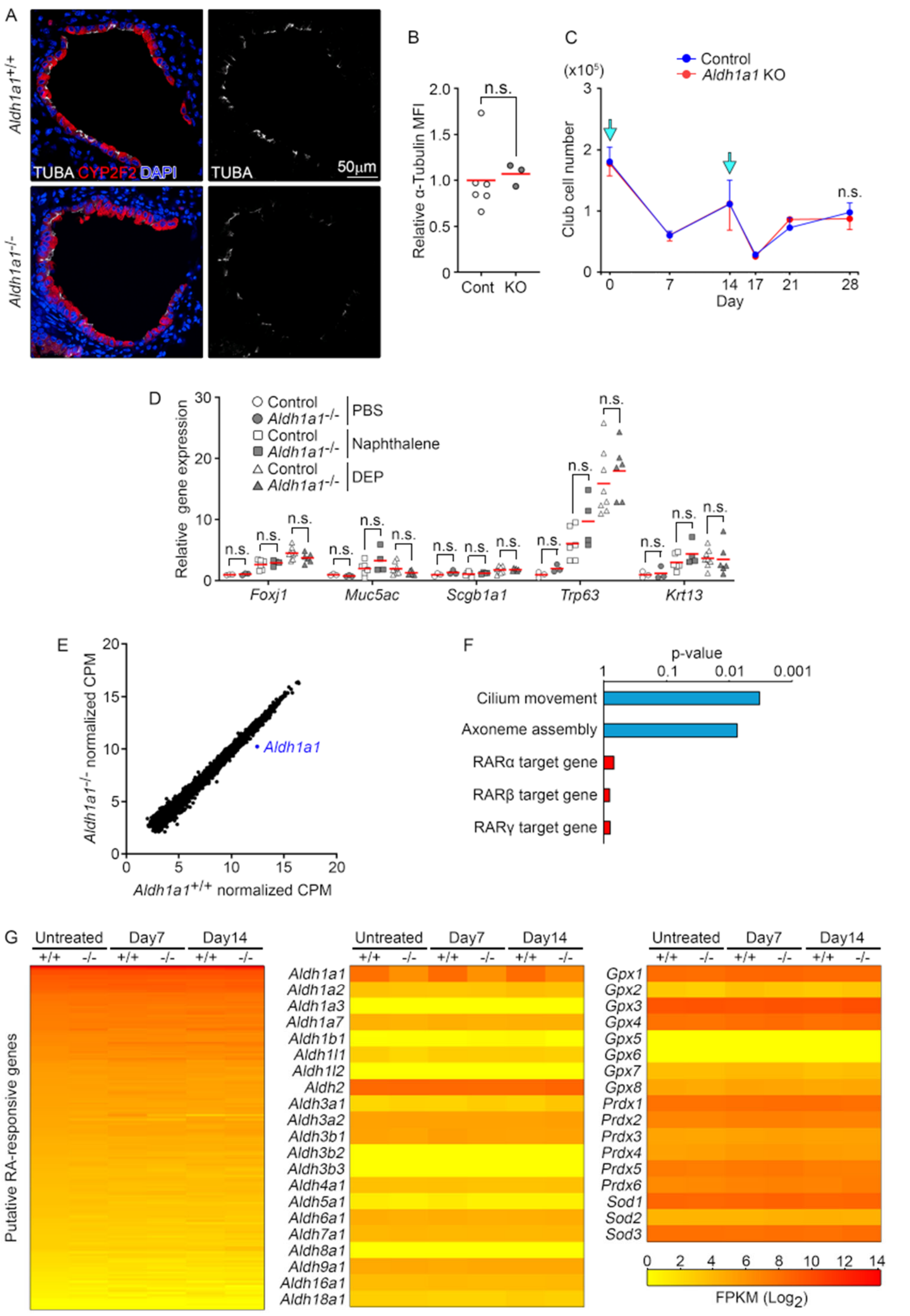

Supplemental Figure 3: ALDH1A1 deficiency leads to aberrant cilia formation

A-C Mice with indicated genotypes were intraperitoneally injected with 200 mg/kg naphthalene at two-week intervals for a total of two times, and lungs were harvested two

weeks after the second injection. **A** Representative images of immunofluorescence staining of longitudinal sections of the small airway epithelium for cilia (TUBA), airway epithelial cells (CYP2F2), and nuclei (DAPI) are shown. **B** Mean fluorescence intensity (MFI) of α-tubulin levels was determined at fourteen days after the last injection of naphthalene (n=3-6). **C** The cell numbers of lung club cells were determined at specified days post administration and the mean values with S.E.M. are shown (n=3-9). The days of naphthalene injection are indicated by light blue arrows. **D** Mice indicated genotypes were intraperitoneally injected with 200 mg/kg naphthalene at two-week intervals for a total of two times. Alternatively, mice were intranasally administered with PBS or 100 μg of DEP every other day for a total of six times. The expression of genes for airway epithelial cell markers in tracheas at fourteen days after the last injection of naphthalene or at three days after the last injection of PBS or DEP was determined (n=3-8). **E** The scatter plots depict the normalized expression levels of transcript abundance in the lungs of *Aldh1a1*$^{+/+}$ and *Aldh1a1*$^{-/-}$ mice, determined by RNA-seq. Expression values represent as $\log_2$ (cpm+c), with a pseudo constant of c=4. The *Aldh1a1* mRNA expression, marded by a blue dot, is reduced in *Aldh1a1*$^{-/-}$ mice, likely due to nonsense-mediated mRNA decay. **F** Biological process terms that exhibit significant differences between *Aldh1a1*$^{+/+}$ and *Aldh1a1*$^{-/-}$ mice in gene ontology enrichment analysis are represented by blue bars. A gene sets for RAR target genes defined in RegNetwork are represented by red bars. **G** *Aldh1a1*$^{+/+}$ and *Aldh1a1*$^{-/-}$ mice were either untreated or intraperitoneally injected with 200 mg/kg naphthalene, and whole lung RNA was determined by RNA-seq at the specified days post administration. Heat maps show the expression of putative RA-responsive genes (left), *Aldh* (middle), and anti-oxidant genes (right). Expression levels are shown as fragments per kilobase of exon per million reads mapped (FPKM, $\log_2$). Data are represented by at least three independent experiments with similar results (A). n.s., not significant by unpaired t-test (B and C) or One-way ANOVA followed by a post-hoc Tukey test (D).

A

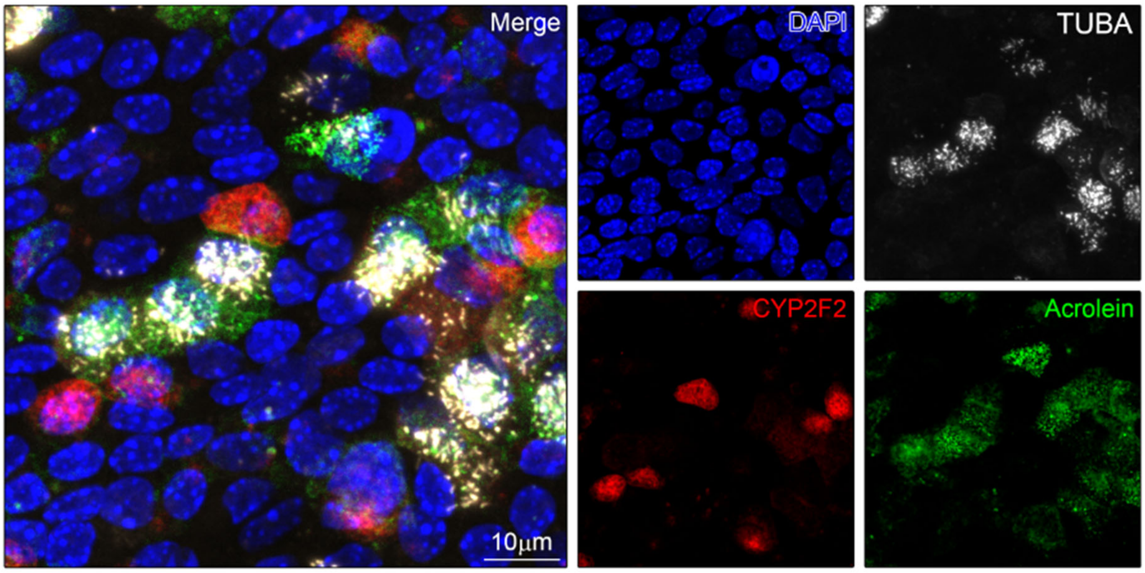

Supplemental Figure 4: Aberrant cilia regeneration in ciliated cell culture

A Immunofluorescence staining of ALI culture was assessed for cilia (TUBA), CYP2F2, Acrolein-adduct, and nuclei (DAPI). Maximum intensity projections of z-stack are presented.

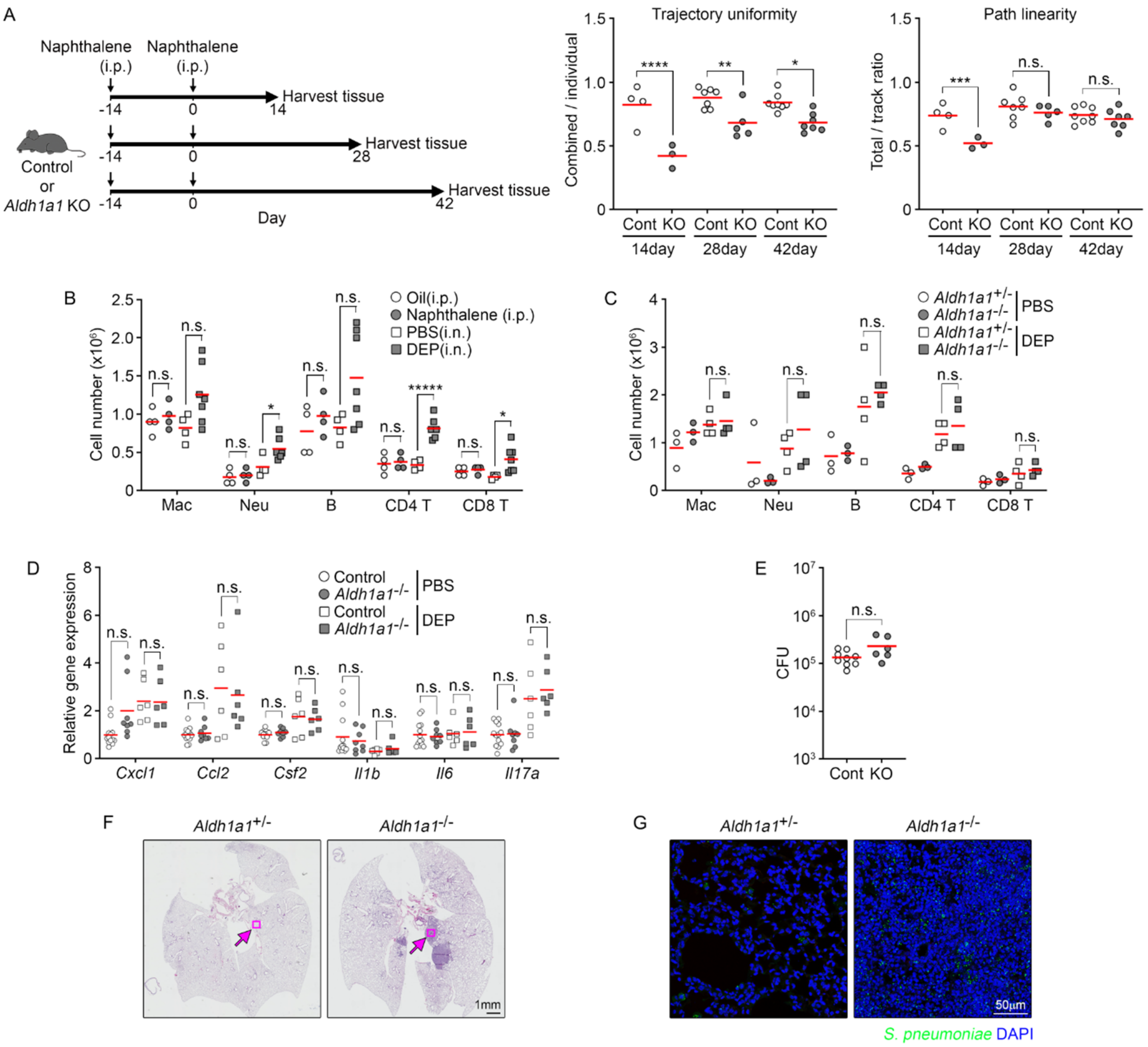

Supplemental Figure 5: Impaired MCC in ALDH1A1-deficient mice

A Mice with indicated genotypes (Cont; *Aldh1a1*$^{+/+}$ or *Aldh1a1*$^{+/-}$, KO; *Aldh1a1*$^{-/-}$) mice were intraperitoneally injected with oil or 200 mg/kg naphthalene at two-week intervals for a total of two times. 14, 28, or 42 days after the second naphthalene injection, mucociliary transport in isolated tracheas were determined using live imaging of fluorescent beads (left). Trajectory uniformity calculated from 10 beads per recorded area (middle) and path linearity of individual beads (right) are presented (n=3-8). **B-D** WT mice were intraperitoneally injected with oil or 200 mg/kg naphthalene at two-week intervals for a total of two times. Alternatively, WT mice were intranasally administered with PBS or 100 μg of DEP every other day for a total of six times. **B** Numbers of macrophages (Mac), neutrophils (Neu), B

cells (B), CD4 T cells (CD4 T), and CD8 T cells (CD8 T) in lungs at fourteen days after the last injection of oil or naphthalene or at three days after the last injection of PBS or DEP were determined (n=4-7). **C** Leukocyte cell numbers in lungs were determined at three days after the last DEP injection are shown (n=3-4). **D** The expression of indicated genes in whole lung tissues was determined by qPCR (n=6-13). **E-G** Mice with indicated genotypes were intraperitoneally injected with oil or 200 mg/kg naphthalene at two-week intervals for a total of two times. two weeks after the second naphthalene injection, mice with were intranasally infected with 1 x 10^8 cfu of *S. pneumoniae*. Three days after *S. pneumoniae* infection, *S. pneumoniae* in the BAL fluid (E) and hematoxylin and eosin (H&E) staining of lungs (F). **G** The regions marked by pink arrows in (F) were determined for immunofluorescence staining for *S. pneumoniae* and nuclei (DAPI). *****P<0.0001, ****P<0.001, ***P<0.01, **P<0.02, *P<0.05, and n.s., not significant by One-way ANOVA followed by a post-hoc Tukey test (A-D) or unpaired t-test (E). Data are represented by at least two independent experiments with similar results (F and G).

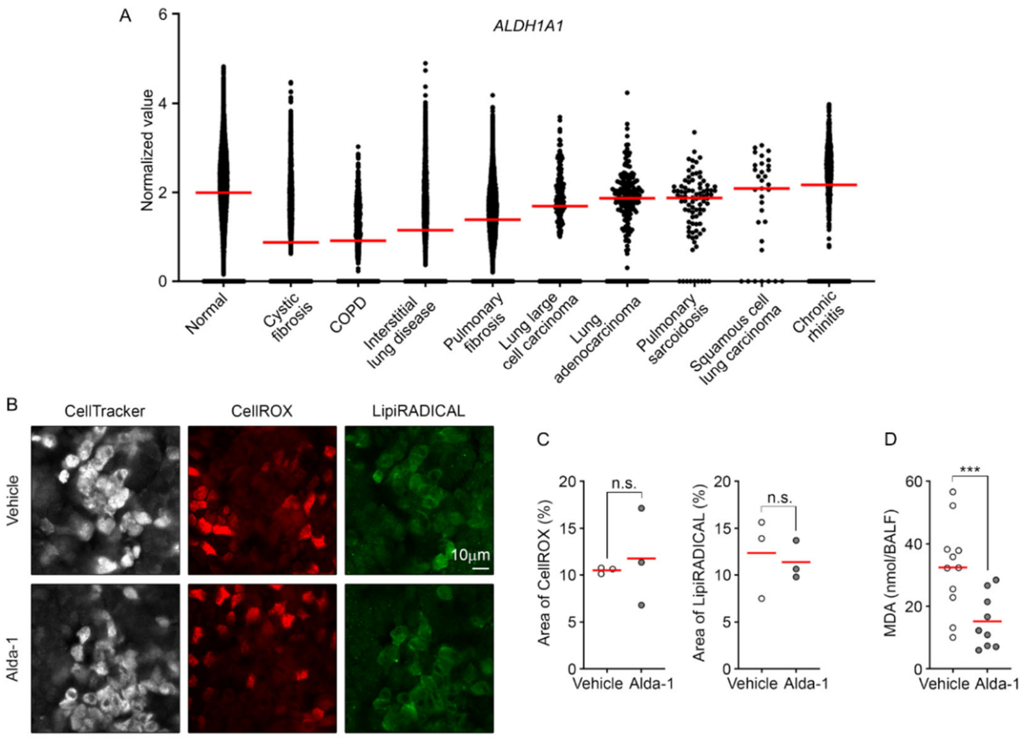

Supplemental Figure 6: Enhancing ALDH1A1 activity promotes regeneration of cilia and MCC

A Plots showing the expression levels of *ALDH1A1* inciliated cells among healthy and patients with respiratory diseases. The median values are shown by red horizontal lines. **B-D** WT mice were infused with vehicle or Alda-1 (0.8mg/kg/h) via implanted osmotic pump. **B** Three days post infusion, mice were intraperitoneally injected with 200 mg/kg naphthalene. For hour later, luminal surface of isolated tracheas was determined for cell shape (CellTracker), oxidative stress (CellROX), and lipid peroxidation (LipiRADICAL). **B** Representative live images are shown. **C** The proportions of the area that were positive for CellROX (left) and LipiRADICAL (right) in (B) were determined (n=3). **D** Two days post infusion, mice were intranasally administered with 100 µg DEP three times per days for two consecutive days. Free-MDA in BALF supernatant was determined eighteen hours after the final administration (n=9-11). Each point represents one mouse and the mean values are shown by red horizontal lines (D). ***P<0.01, and n.s., not significant by unpaired t-test. Data are represented by at least two independent experiments with similar results (B and C).
