# Supplementary figures and images for: Aldehyde metabolism governs resilience of mucociliary clearance to air pollution exposure
Source: J Clin Invest. 2025 May 15;135(14):e191276. doi: 10.1172/JCI191276 (PMC12259252; doi:10.1172/JCI191276)

**Supplemental Figure 2A**

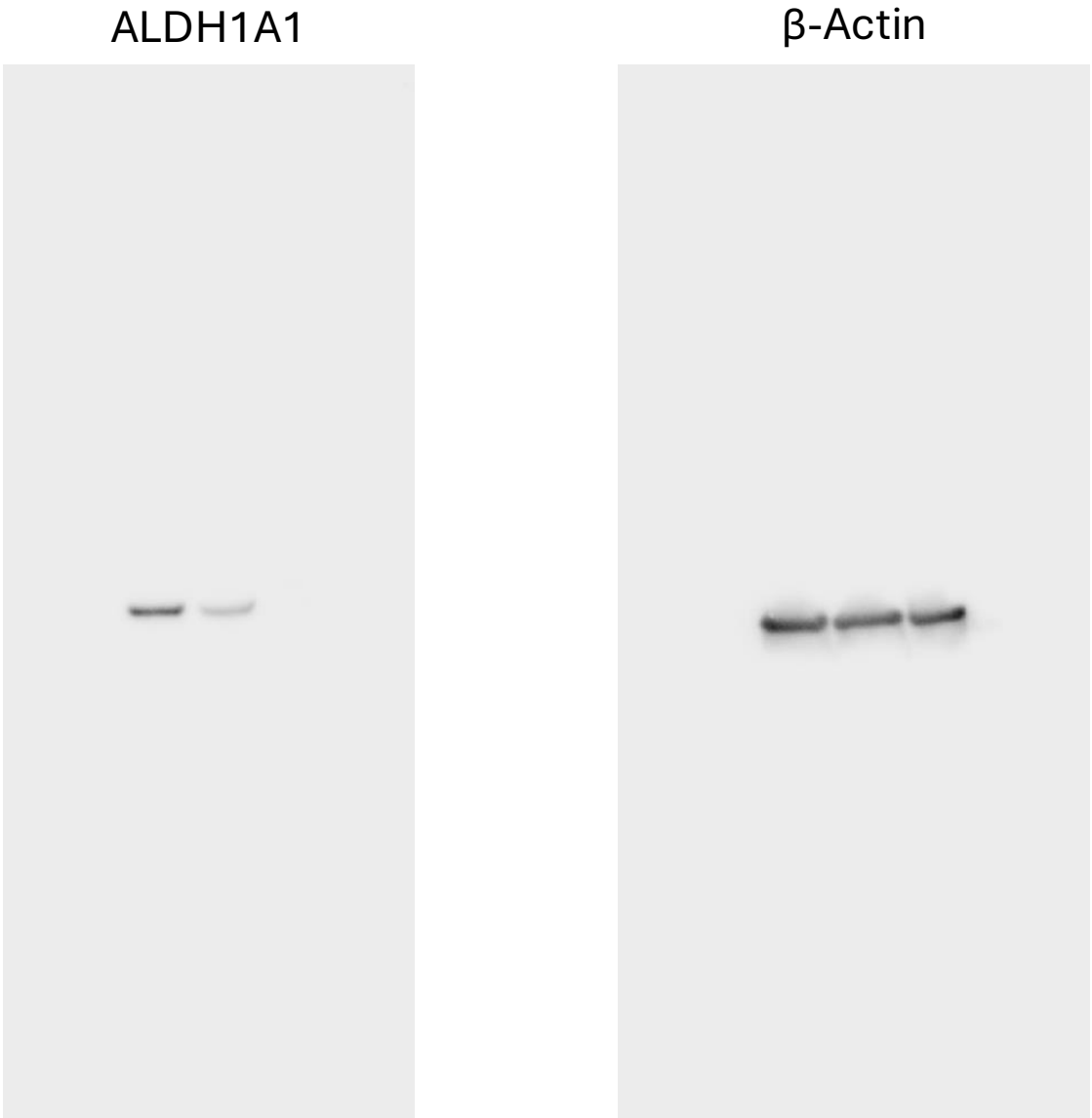

Figure 6D

TUBA

$\beta$ -Actin

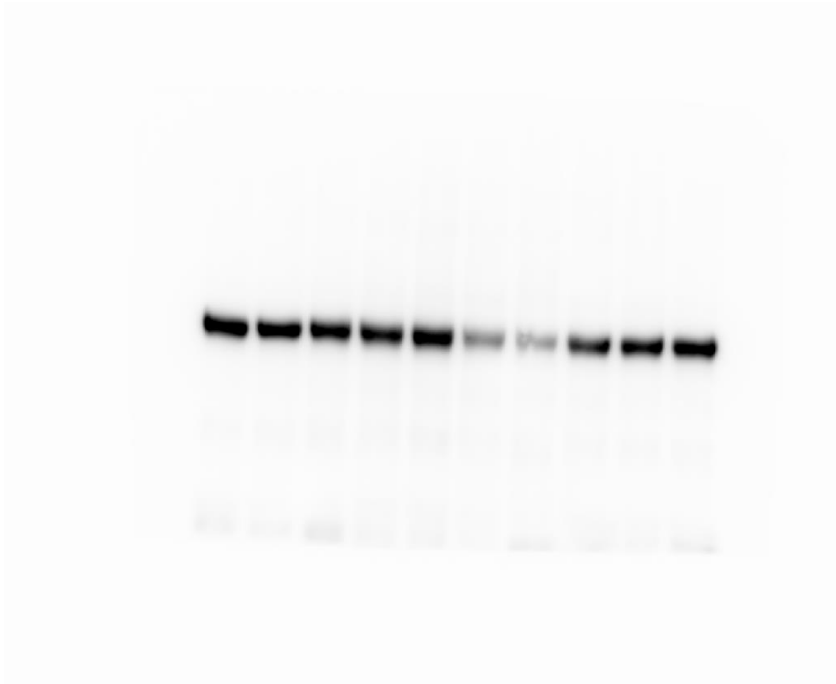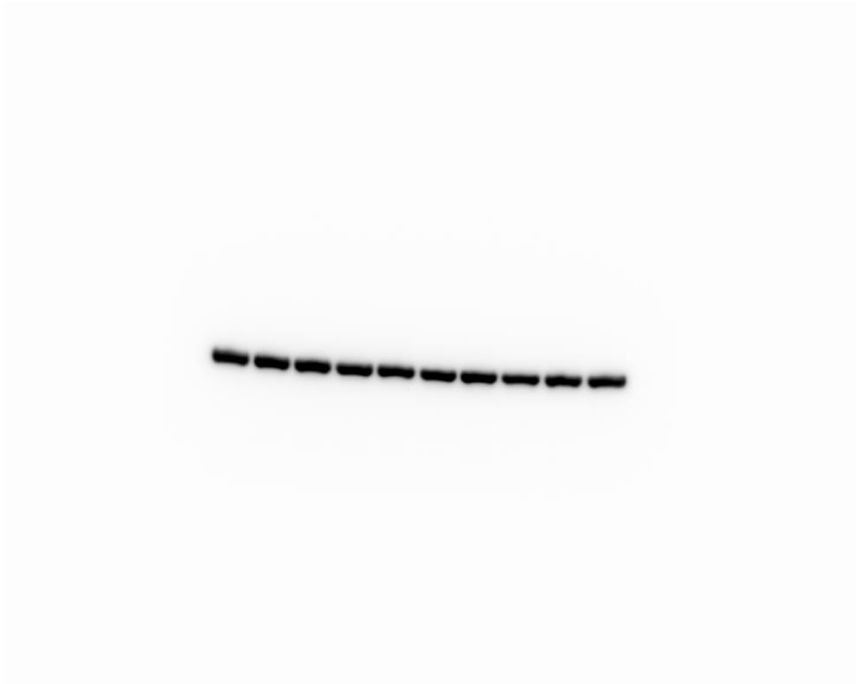

Supplement: Unedited blot and gel images [file jci-135-191276-s104.pdf]
